# Placental Macrophage (Hofbauer Cell) Responses to Infection During Pregnancy: A Systematic Scoping Review

**DOI:** 10.3389/fimmu.2021.756035

**Published:** 2022-02-11

**Authors:** Georgia Fakonti, Paschalia Pantazi, Vladimir Bokun, Beth Holder

**Affiliations:** Institute of Reproductive and Developmental Biology, Department of Metabolism, Digestion and Reproduction, Imperial College London, London, United Kingdom

**Keywords:** Hofbauer cells, placental macrophages, placenta, congenital infection, virus

## Abstract

**Background:**

Congenital infection of the fetus *via* trans-placental passage of pathogens can result in severe morbidity and mortality. Even without transmission to the fetus, infection of the placenta itself is associated with pregnancy complications including pregnancy loss and preterm birth. Placental macrophages, also termed Hofbauer cells (HBCs), are fetal-origin macrophages residing in the placenta that are likely involved in responding to placental infection and protection of the developing fetus. As HBCs are the only immune cell present in the villous placenta, they represent one of the final opportunities for control of infection and prevention of passage to the developing fetus.

**Objective and Rationale:**

The objective of this review was to provide a systematic overview of the literature regarding HBC responses during infection in pregnancy, including responses to viral, bacterial, and parasitic pathogens.

**Methods:**

PubMed and Scopus were searched on May 20th, 2021, with no limit on publication date, to identify all papers that have studied placental macrophages/Hofbauer cells in the context of infection. The following search strategy was utilized: (hofbauer* OR “hofbauer cells” OR “hofbauer cell” OR “placental macrophage” OR “placental macrophages”) AND [infect* OR virus OR viral OR bacteri* OR parasite* OR pathogen* OR LPS OR “poly(i:c)” OR toxoplasm* OR microb* OR HIV)].

**Outcomes:**

86 studies were identified for review. This included those that investigated HBCs in placentas from pregnancies complicated by maternal infection and *in vitro* studies investigating HBC responses to pathogens or Pathogen-Associated Molecular Patterns (PAMPs). HBCs can be infected by a variety of pathogens, and HBC hyperplasia was a common observation. HBCs respond to pathogen infection and PAMPs by altering their transcriptional, translational and secretion profiles. Co-culture investigations demonstrate that they can replicate and transmit pathogens to other cells. In other cases, they may eliminate the pathogen through a variety of mechanisms including phagocytosis, cytokine-mediated pathogen elimination, release of macrophage extracellular traps and HBC-antibody-mediated neutralization. HBC responses differ across gestation and may be influenced by pre-existing immunity. Clinical information, including gestational age at infection, gestational age of the samples, mode of sample collection and pregnancy outcome were missing for the majority of studies.

## Introduction

The maternal-fetal interface is the site of *in utero* pathogen transmission from mother to the fetus during pregnancy. The maternal decidua, which is in close contact with the placenta, contains several immune cell types including macrophages, natural killer cells, innate lymphoid cells and T cells. In contrast, the only immune cells present in placental tissue are macrophages termed Hofbauer cells (HBCs). These macrophages are of fetal origin and have been detected from the sixth week of pregnancy to the end of gestation (1). HBCs help maintain homeostatic conditions in the placenta by regulating processes such as angiogenesis, vasculogenesis (2, 3), tissue remodeling and (4) development (5) and immune regulation (6), as well as orchestrating responses to infection and directly responding to pathogens that access the placenta. The functional variability of HBCs reflects the phenotypic diversity and plasticity of macrophages and allows adaptation to different microenvironments (7–10).

Pathogens can be transmitted from mother to fetus either *via* infection of the placenta or fetal membranes (often viral), ascension of the reproductive tract (often bacterial) or by transmission during childbirth (viral, bacterial, fungal). Infection that occurs whilst the baby is *in utero* is termed congenital infection. Each year congenital anomalies lead to approximately 300,000 neonatal deaths during the first month of their lives (11). Globally, approximately 190,000 cases of congenital toxoplasmosis (12) and 1,000,000 incidences of congenital syphilis (13) are reported each year. Pathogens capable of causing congenital infection have historically been classified as members of the ‘TORCH complex’: **T**oxoplasma gondii, **O**thers (syphilis, Hepatitis B), **R**ubella, **C**ytomegalovirus (CMV) and **H**erpes simplex virus (HSV) (14). Other pathogens such as Zika virus (ZIKV), Human Immunodeficiency Virus (HIV), Varicella-zoster virus (VZV), *Listeria monocytogenes* and *Treponema pallidum* have subsequently been added to this group (15). Congenital infection can be associated with considerable morbidity and mortality, depending on the developmental stage of the fetus at the time of infection and the causative pathogen (16, 17). Consequences include neurodevelopmental delays, hearing loss (18), microencephaly and other major neurological abnormalities (19).

Placental infection with or without concomitant pathogen transmission to the fetal compartment can cause distinct histopathological findings in the placental tissue. Specifically, infectious processes in the placenta can be accompanied by villitis, a destructive inflammation of the chorionic villi associated with lower birth weight (20), or chorioamnionitis (CA), inflammation of the fetal membranes often associated with spontaneous preterm birth (21). Evidence accumulated since the start of the COVID-19 pandemic indicates that SARS-CoV-2 could be an example of a pathogen capable of infecting the placenta but not readily transferred transplacentally to the fetus in most cases (22, 23). SARS-CoV-2 protein and RNA have been detected in the syncytiotrophoblast cells and less so in other cell types in the placenta, while marked inflammation and lymphohistiocytic infiltration were noted as a consequence of placental infection (24–26). Whilst the presence of pronounced inflammation in this case can be damaging to the placental tissue, it could be essential for preventing further SARS-CoV-2 spread and subsequent *in utero* infection, a scenario with a worse potential outcome. Several published studies of placental SARS-CoV-2 infection are lacking in their methodology and terminology; the importance of proper sampling and techniques for accurate definition of placental SARS-CoV-2 infection was recently published (27).

Given the significant medical, societal and economic impacts of placental and fetal infection in pregnancy (28, 29), and the easy accessibility to these tissue macrophages at the end of pregnancy, it is surprising that we do not better understand the roles that Hofbauer cells play in infective processes. This information can help in the establishment of new preventive strategies and therapeutic interventions to protect individuals at a very vulnerable stage of the life course. There are several excellent previous reviews of HBC biology and their role in normal and pathological pregnancy (10, 28, 29). This scoping review follows systematic review methodology to synthesize the literature specifically regarding the role of HBCs in infection during pregnancy.

## Methods

### Search Strategy

Studies without any publication year limit were retrieved from PubMed and Scopus on 20^th^ May 2021 using a combination of key words designed to capture all articles that have investigated HBCs and infection. The keywords were selected to be in the title/abstract for PubMed and title/abstract/keywords for Scopus search to identify results focused on the main research question. The search was as follows: (hofbauer* OR “hofbauer cells” OR “hofbauer cell” OR “placental macrophage” OR “placental macrophages”) AND [infect* OR virus OR viral OR bacteri* OR parasite* OR pathogen* OR lps OR “poly (i:c)” OR toxoplasm* OR microb* OR HIV)]. Additional keywords were tested without identifying any additional studies, and therefore were excluded from the final search (**Supplementary Material - Table 1**).

### Inclusion and Exclusion Criteria

Only studies that used human material from infected patients and investigated HBCs responses were included. Studies that investigated HBCs (isolated from human placenta) in combination with pathogens or pathogen-associated molecular patterns (PAMPs) were also included. Excluded criteria included (a) usage of animal models or cell lines without the use of any primary human placental macrophages (b) reviews and book chapters (c) studies not written in English. Duplicates found during title/abstract screening or/and full-text screening and studies without full text available were also excluded (**Supplementary Material - Table 2**).

### Study Screening

**Figure 1:**
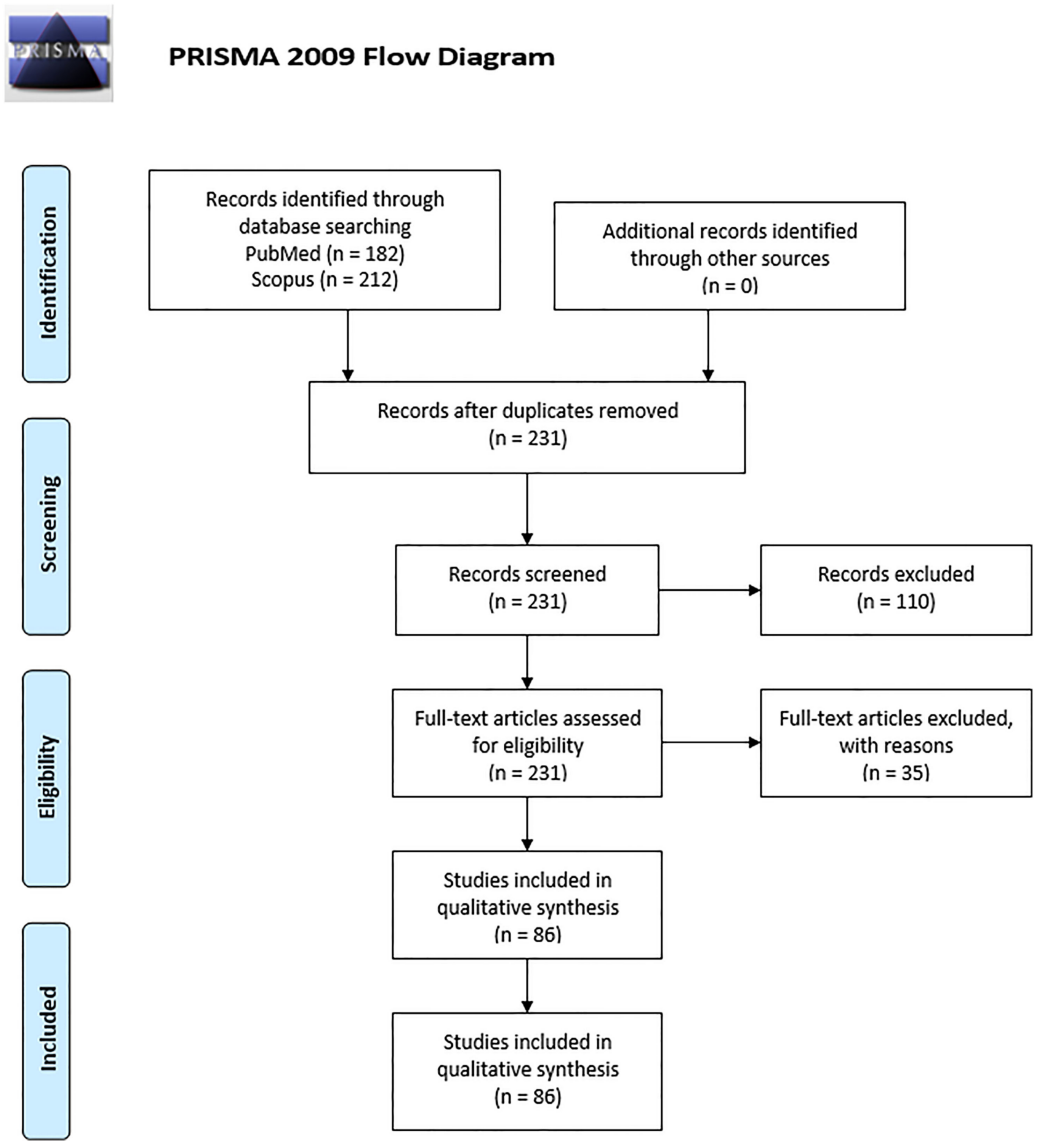
PRISMA 2009 flow diagram of literature search and records selection.

For article screening and Preferred Reporting Items for Systematic Reviews and Meta-Analyses (PRISMA) flow diagram creation (**Figure 1**), the Covidence systematic review platform was used. The articles were screened independently by two reviewers (G. F. and P. P.) for eligibility. A third reviewer (B. H.) resolved any conflicts that arose during the screening. For the purposes of this review the risk of bias for each study was not evaluated since no cohort studies nor randomized control trials were present.

### Data Extraction and Analysis

To facilitate the interpretation of the final list of results, the following data were extracted from the articles: article identification (title, authors, journal and publication year), study design (case-control, *in vitro*), health status of the samples (normal pregnancy, villitis, CA and preeclampsia), mode of sample collection (birth, termination, miscarriage and stillbirth), gestational age (first, second or third trimester), sample type (placental tissue, explants and isolated HBCs), method of HBC isolation (digestion and centrifugation, positive selection with anti-CD14 immunobeads, negative selection using anti-CD10 and anti-epidermal growth factor receptor (EGFR) immunobeads or other), sample treatment (co-culture, *in vitro* exposure to pathogen or PAMPs), methods to identify infection, placental infection prevalence, fetal infection, HBC infection, HBC hyperplasia, gene transcription and protein expression or secretion and the main findings are summarized in tables across results section.

## Results

### Studies Identified

**Figure 2:**
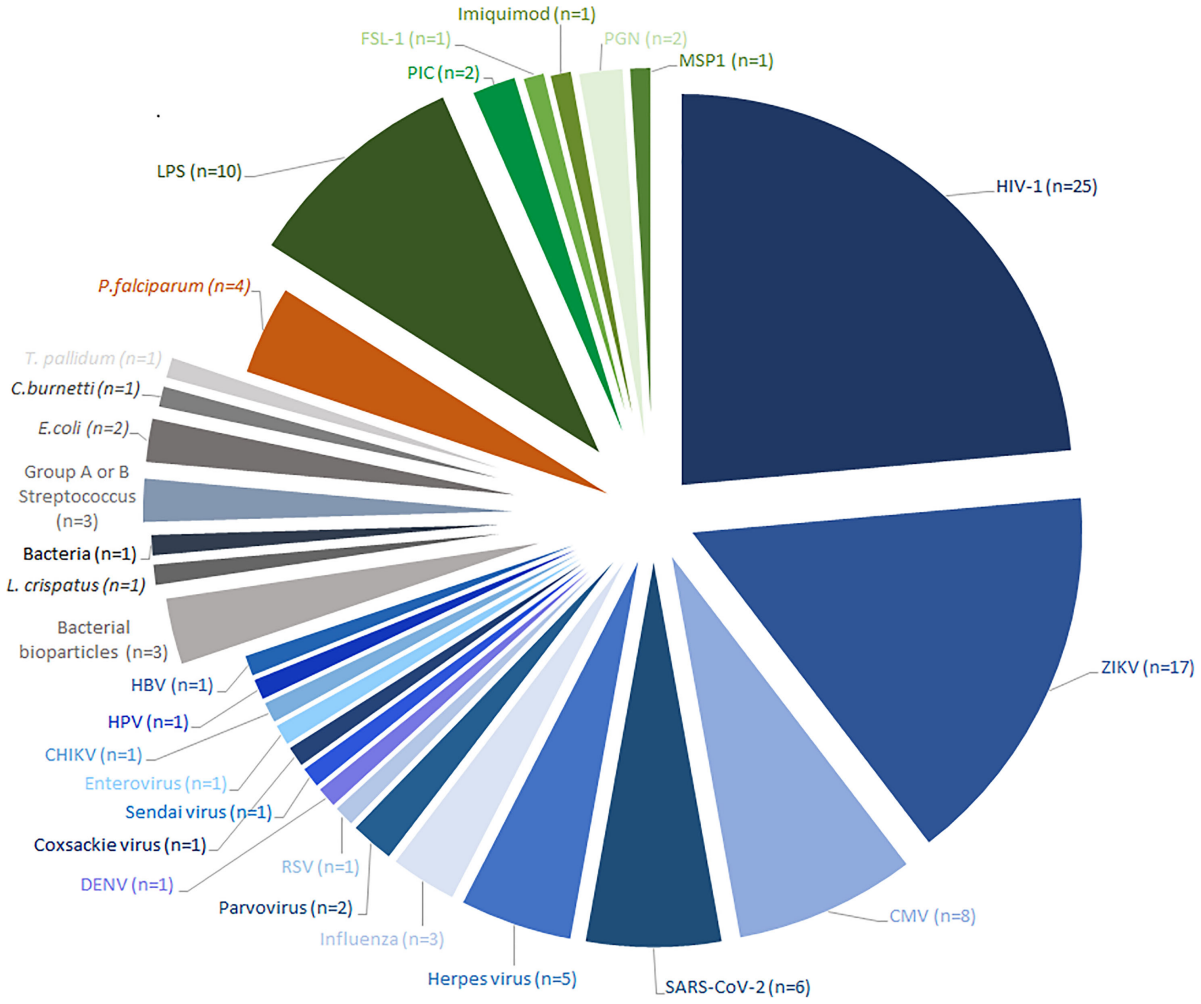
Overview of all studies that have investigated pathogen infection or pathogen responses in Hofbauer cells. All identified studies published before 20^th^ May 2021 were categorized by pathogen, or Pathogen-Associated Molecular Pattern (PAMP) studied. Viral infections are represented in blue, bacterial infections in grey, parasitic infections in orange and PAMP treatments in green. The number of articles that investigated each pathogen or PAMP is indicated in parentheses. HIV Human Immunodeficiency Virus, ZIKV Zika virus, CMV Cytomegalovirus, RSV Respiratory syncytial virus, DENV Dengue virus, CHIKV Chikungunya virus, HPV Human papillomavirus, HBV Hepatitis B virus, L. crispatus Lactobacillus crispatus, E.coli Escherichia coli, T.pallidum Treponema pallidum, C.burnetti Coxiella burnetii, P.falciparum Plasmodium falciparum, LPS lipopolysaccharide, PIC Polyinosinic:polycytidylic acid, FSL-1 Pam2CGDPKHPKSF-synthetic lipopeptide, PGN Peptidoglycan, MSP1 Merozoite surface protein.

From a total of 231 studies initially screened, 86 were included (**Figure 1**). Of these, 37 were *in vitro* studies using isolated HBCs or explants, 47 were observational studies using placental tissue, which included case-control studies and case reports and 2 studied both. The results were divided into 4 main groups based on the type of infection; HBCs in: 1) viral infection (n=67) including infections by HIV (n=26), ZIKV (n=17), CMV (n=8) and others; 2) bacterial infection (n=7); 3) parasitic infection, which were all studies of *Plasmodium falciparum* (n=4); or 4) response to Pathogen-Associated Molecular Patterns (PAMPs) (n=13). An overview of the study of HBCs and infection is shown in **Figure 2**.

### HBCs in Viral Infection

#### HIV-1 Observational Studies

HIV-1 was the most investigated pathogen in observational studies (n=6/9) (30–35). Placentas were obtained from mothers after pregnancy termination, pregnancy loss or delivery (**Table 1**). HIV-1 infection of the placenta was detected in 27% to 100% of samples. Infection of HBCs was assessed through observation of viral protein (n=5), reporting 30% - 100% of samples with infected HBCs and through detection of viral nucleic acid (n=4), reporting infected HBCs in 16% to 100% patients (**Table 1**). The proportion of infected HBCs in each placenta was missing from most studies; however, Lewis et al. (30) reported that one in five HBCs were positive for HIV-1 and Backé et al. (33) reported the presence of a few positive HBCs.

**Table 1:** Hofbauer cells in observational studies of viral infection.

Virus	Samples studied by trimester (outcome)				HBC infection diagnosis method	Key outcome(s) in HBCs		Reference
	1^st^	2^nd^	3^rd^ <37w	3^rd^ ≥37w		HBC infection detected (no.)	HBC hyperplasia (%)	
**HIV-1**	3 (TOP)	–	–	–	Protein & RNA	+ (3/3 protein) & + (2/3 nucleic acid)	NR	Lewis et al. (30)
	23 (TOP) gestational age between 6-39w & 11 term (LB)				Protein	+ (10/23 & 5/11)	NR	Backé et al. (31)
	9 gestational age NR (NR)				Protein	+ (4/9)	NR	Martin et al. (32)
	48 gestational age NR (LB)				Protein & RNA	+ (NR)	NR	Backé et al. (33)
	–	–	–	23 (LB)	NR	NR	NR	Behbahani et al. (36)
	–	–	2 (LB)	1 (LB)	RNA	+ (3/3)	NR	Sheikh et al. (34)
	24; 8-20w (TOP)		–	–	Protein & RNA	+ (8/24 protein) & + (4/24 nucleic acid)	NR	Bhoopat et al. (35)
	–	–	–	40 (LB)	NR	NR	NR	Pillay et al. (37)
	–	–	–	99 (LB)	NR	NR	NR	Martinez et al. (38)
**ZIKV**	1 (PL)	–	–	2 (LB)	Protein	+ (1/1)	+ (100%)	de Noronha et al. (39)
	–	1 (TOP)	–	–	RNA	+ (1/1)	+ (100%)	Rosenberg et al. (40)
	–	1 (TOP)	1 (PL)	–	RNA	+ (1/1)	+ (100%)	Schwartz, (41)
	1 (PL)	–	3 (LB)	19 (LB) & 1 (PL)	Protein	+ (NR)	+ (35%)	de Noronha et al. (42)
		1 (PL)	–	–	Protein	+ (1/1)	+ (100%)	Rabelo et al. (43)
	–	3 (TOP)	–	–	NR	NR	+ (100%)	Beaufrère et al. (44)
	–	–	–	3 (LB)	Protein	+ (3/3)	- (0%)	Lum et al. (45)
	–	–	–	1 (LB)	NR	NR	+ (100%)	Santos et al. (46)
	–	–	4 (LB)	–	NR	NR	+ (100%)	Miranda et al. (47)
**CMV**	–	1 (PL)	1 (PL) & 3 (LB)	1 (PL)	Protein	–	NR	Mühlemann et al. (48)
	–	3 (PL)	1 (LB & neonatal death)	–	Protein	–	+ (100%)	Schwartz et al. (49)
	–	1 (PL)	4 (3 LB & 1 PL)	1 (PL)	Protein	–	NR	Mühlemann et al. (50)
	3 gestational age NR (NR)				DNA	+ (NR)	+ (100%)	Euscher et al. (51)
	–	–	4 between 28-41w (PL or/and LB & neonatal death)		DNA	+ (NR)	NR	Satosar et al. (52)
**Coxsackie virus**	–	6 cases gestational age 25-43w (LB)			Protein + RNA	+ (NR) & + (NR)	+ (NR)	Euscher et al. (51)
**HSV**	1 case of infection gestational age NR (NR)				DNA	+ (1/1)	- (0%)	Euscher et al. (51)
	–	–	2 cases between 28-41w (PL or/and LB)		DNA	+ (NR)	NR	Satosar et al. (52)
**Enterovirus**	–	–	23 cases between 28-41w (PL or/and LB & neonatal death)		Protein & RNA	+ (12/23) & + (NR)	NR	Satosar et al. (52)
**Parvovirus**	–	–	2 cases between 28-41w (PL or/and LB & neonatal death)		DNA	- (NR)	NR	Satosar et al. (52)
	1 gestational age NR (PL)				DNA	- (1/1)	+ (100%)	Euscher et al. (51)
**H5N1**	–	1 (PL)	–	–	NR	NR	NR	Yao et al. (53)
	–	1 (PL)	–	–	Protein & RNA	+ (1/1)	NR	Gu et al. (54)
**HBV**	–	–	–	28 (LB)	NR	NR	NR	Liu et al. (55)
**HPV**	100 (54 TOP + 46 PL)		68 (LB)	103 (LB)	DNA	+ (1/1)	NR	Ambühl et al. (56)
**DENV**	–	–	1 (PL)	–	Protein	+ (1/1)	+ (100%)	Nunes et al. (57)
**Chikungunya virus**	4 (PL)		–	–	Protein	+ (3/4)	NR	Salomão et al. (58)
**SARS-CoV-2**	–	–	–	1 (LB)	Protein & RNA	+ (1/1)	+ (100%)	Facchetti et al. (26)
	–	1 (PL)	8 (LB)	10 (LB)	NR	NR	+ (5.5%)	Hecht et al. (25)
	–	–	–	1 (LB)	Protein & RNA	- (1/1)	+ (100%)	Morotti et al. (59)
	–	1 (LB)	–	4 (LB)	Protein & RNA	+ (5/5)	NR	Verma et al. (60)
	–	–	1 (LB)	7(LB)	Protein & RNA	- (8/8)	- (100%)	Gao et al. (61)

TOP, Termination of pregnancy; LB, Live birth; NR, Not reported; PL, Pregnancy loss (stillbirth/miscarriage); HIV-1, Human immunodeficiency virus 1; ZIKV, Zika virus; CMV, Cytomegalovirus; HSV, Herpes simplex virus; H5N1, Influenza virus A; subtype H5N1; HBV, Hepatitis B virus; HPV, human papilloma virus; DENV, Dengue virus; SARS-CoV-2, Severe acute respiratory syndrome coronavirus 2.

Four studies examined HBC infection and transplacental transmission (30, 33, 34, 37). HIV-1 was detected in embryonic precursor blood cells in three first trimester fetuses with infected HBCs, underlying a possible role of HBCs in fetal infection during early gestation (30). Backé et al. (33) reported 12 asymptomatic offspring from 13 HIV-1 antigen-positive placentas and one infected offspring from four HIV-1-positive placentas. Sheikh et al. (34) reported three cases of HIV-1 *in utero* transmission in women with syphilis, one of them also having chorioamnionitis, and observed that HBC infection indicated a possible involvement of HBCs in offspring infection during late pregnancy. Cases of chorioamnionitis were reported in three studies (34, 35, 37); however, only the study of Bhoopat et al. (35) investigated the contribution of chorioamnionitis in vertical transmission of HIV-1. In this study, 24 first and second trimester therapeutic abortions from HIV-1 seropositive women were examined and placental histological analysis revealed five cases of chorioamnionitis (35). Of these five cases, four were associated with fetal viral transmission, suggesting a link between HBC infection, placental inflammation and HIV-1 in vertical transmission during early pregnancy.

The three remaining observational studies of HIV in HBCs were focused on proteins associated with the immune response or viral entry. One study investigated the placental expression of HIV-1 co-receptors, C-C chemokine receptor type 5 (CCR5) and C-X-C chemokine receptor type 4 (CXCR4) in 16 non-transmitting and 7 transmitting HIV-1 women (36). They reported expression of both co-receptors in HBCs but not in trophoblast cells and identified that placental CCR5:CXCR4 ratio was significantly higher in transmitting women; however, they did not further investigate whether the alteration was due to upregulation or downregulation of co-receptors in HBCs. Also, higher expression levels of DC-SIGN and DC-SIGNR, lectins that recognize viral PAMPs, were reported in placentas from 25 women with HIV-1 and 15 women with HIV-1 and CA compared to HIV-negative women, and HBCs were reported to express both proteins (37). No difference in the expression of the natural killer inhibitory receptor ligand, HLA-E, was documented in placental tissue from 99 HIV-infected mothers compared to uninfected group in another study (38). Though HLA-E was expressed in HBCs, differences between HIV-positive and negative women were not examined.

#### HIV-1 *In Vitro* Studies

Fifteen studies examined the permissiveness of HBCs to HIV-1 using *in vitro* challenge (**Table 2**). Of these, infection was reported through detection of HIV-1 nucleic acid (34, 62, 63, 67, 68, 73, 75, 77) or protein (62, 65, 66, 70, 73, 76). Productive infection was also measured by extracellular protein (62–66, 68, 69, 71, 75, 77), extracellular reverse transcriptase activity (62), infectious virions in supernatant (62, 65) or/and microscopy (75, 76). Of the 17 studies, eight investigated susceptibility to HIV-1 infection in HBCs compared to monocytes or monocyte-derived macrophages (MDMs) through p24 detection in supernatants (n=8) or/and viral nucleic acid (n=5) (62, 63, 69, 73, 75). These studies reported HIV-1 replication in HBCs but reduced ability to replicate the virus compared to fetal MDMs, adult blood and cord MDMs. Even upon GM-CSF treatment, HBCs produced fewer virions compared to fetal MDMs (62) and adult MDMs (64). The distinct difference in viral replication was not due to increased cell death, cytopathic effects (62, 64, 68, 75) or inefficient particle assembly/release (75), but due to restriction at the transcriptional level (73).

**Table 2:** Hofbauer cells in *in vitro* studies of viral infection.

Virus	Samples studied by trimester (outcome)						Model(s) of infection	Immuno-purification of HBCs	Diagnosis of HBC infection/ viral production (no.)	Key outcome(s) in HBCs	Reference
	1^st^	2^nd^	3^rd^ <37w		3^rd^ ≥37w						
**HIV-1**	3 (NR)	3 (NR)					Cells & co-culture	No	RT activity, antigen release, virion release, PCR-Southern & IF	Productive HIV-1 infection	Mano and Chermann (62)
	–	–		–		NR (LB)	Cells	No	ISH & antigen release	Productive HIV-1 infection. HIV-1 clinical isolates exhibit different tropism	Kesson et al. (63)
	NR						Cells	No	Antigen release	Infection peak at 3-7 days with no subsequent rise up to 60 days. HIV-1 replication in HBCs not affected by PHA-PBMCs nor treatment with GM-CSF or TNF-α	Kesson et al. (64)
	–	–	–		5 (LB)		Cells	No	Antigen release, infectious virions in supernatant & IF	Different HIV-1 replication rates between HIV-1 strains and HBC donors	McGann et al. (65)
	–	–	–		NR (LB)		Cells	No	IF & antigen release	HBC displayed a similar increase in p24 as adult and cord blood monocytes in response to HIV-1_baL_ monocyte-tropic strain	Meléndez-Guerrero et al. (66)
	–	–	–		NR (LB)		Cells	No	PCR	Zidovudine and progesterone suppress HIV-1 replication in HBCs	Lee et al. (67)
	–	–	–		NR (LB)		Cells	No	PCR & antigen release	Varying susceptibility to different HIV-1 strains and isolates	Fear et al. (68)
	–	–	–		NR (LB)		Cells	No	Antigen release	Altered cytokine secretion	Plaud-Valentin et al. (69)
	–	–	–		6 (LB)		Explants	No	IS-PCR	Infection of HBCs in placental explants	Sheikh et al. (34)
	–	–	–		5 (LB)		Cells & co-culture	No	IF	Infected syncytiotrophoblast transmits the virus to HBCs which release cytokines that induce HIV-1 replication	Bácsi et al. (70)
	–	–	–		4 (LB)		Cells	No	Antigen release	Different HIV-1 replication and viral protein abundance in HBCs and MDMs	Luciano-Montalvo et al. (71)
	–	–	–		4 (LB)		Cells	No	NR	The levels of STAT-1-tyr phosphorylation are lower in HIV-1 infected HBCs	Luciano-Montalvo and Meléndez (72)
	–	–	–		3 (LB)		Cells	No	PCR & Western blot	Lower HIV-1 replication in HBCs compared to MDMs due to restricted transcription	García-Crespo et al. (73)
	–	–	–		9 (LB)		Cells	No	NR	DC-SIGN promoter variants in HBCs influence HIV-1 transmission from mother to child	Boily-Larouche et al. (74)
	–	–	–		40 (LB)		Cells & co-culture	CD14^pos^	Antigen release, PCR & electron microscopy	HBCs display reduced replication and ability to transmit HIV-1_baL_ to PBMCs compared to MDMs; stimulation of HBCs with IL-10, TGF-β, IFN- γ reduces infection in HBCs	Johnson and Chakraborty (75)
	–	–	–		20 (LB)		Cells	CD14^pos^	IF & electron microscopy	HIV-1 assembles in VCCs accessible to neutralizing antibodies which reduce viral replication *via* FcγRI	Johnson et al. (76)
	–	–	–		10 (LB)		Cells	CD14^pos^	Antigen release & PCR	CMV infected HBCs enhance HIV-1 replication in HBCs	Johnson et al. (77)
**ZIKV**	26 (TOP)	–	–		–		Cells & explants	CD14^pos^	RNA (6) & antigen (4)	ZIKV infects HBCs and damages the placenta architecture	El Costa et al. (78)
	–	–	–		3 (LB)		Cells & explants	CD10^neg^/EGFR^neg^	RNA (3) & antigen (3)	ZIKV infects HBCs *in vitro*	Jurado et al. (79)
	4 (TOP)	–	–		–		Explants	N/A	Antigen (4)	ZIKV infects HBCs *in vitro* and expresses important viral entry cofactors	Tabata et al. (80)
	–	–	–		5 (LB)		Cells	CD14^pos^	RNA, antigen & plaque assay (5)	ZIKV can replicate in HBCs with infection rate and antiviral response varying among donors	Quicke et al. (81)
	7 (TOP)	–	–		–		Explants	N/A	Antigen (6)	HBC infection rate varies among strains and donors	Tabata et al. (82)
	–	–	–		12 (LB)		Cells	CD14^pos^	RNA & antigen	JAK-STAT signalling influences the ability of HBCs to produce mature virions	Gavegnano et al. (83)
	–	4 (TOP)	–		–		Cells & explants	CD14^pos^	RNA, antigen & plaque assay (3)	Pre-existing DENV antibodies enhance the HBC infection with ZIKV	Zimmerman et al. (84)
	3 (NR)	–	–		–		Explants	N/A	Antigen	ZIKV NS1 induced shedding of HA and HS, altered expression of CD44 and LYVE-1, and increased placental explant permeability	Puerta-Guardo et al. (85)
**CMV**	–	–	–		5 (LB)		Cells & co-culture	No	Antigen	IL-8 and TGF-1β released upon HBC-syncytiotrophoblast contact stimulates CMV replication in the STB	Bácsi et al. (86)
	–	–	–		NR (LB)		Cells	CD14^pos^	Antigen	Poxvirus-based vaccine-induced neutralizing antibodies prevent CMV infection of HBCs	Wussow et al. (87)
	–	–	–		10 (LB)		Cells & co-culture	CD14^pos^	GFP positive cells	CMV induces TNF-α and IL-6 secretion and supresses STAT2 phosphorylation to supress type I interferon response	Johnson et al. (77)
**HSV-2**	6 (LB)						Cells	No	IF & viral titre measurement	HBCs are not very permissive to HSV and do not support productive replication	Plaeger-Marshall et al. (88)
**HSV-2**	Number and gestational age NR (LB)						Cells	No	IF, viral titration & microscopy (NR)	HBCs prevent HSV-2 and echovirus-type 19 infection	Oliveira et al. (89)
**Echovirus-type 19**
**Sendai virus**	–	–	36 – 42w (LB)				Cells	No	Quantify viral concentration in culture media	Sendai virus induces HBC IFN-β secretion	Toth et al. (90)
**RSV**	–	–	–		5 (LB)		Cells	CD10^neg^/EGFR^neg^	IF, PCR & Western blot (5)	HBCs are permissive to RSV infection and can transfer the virus to neighbouring cells	Bokun et al. (91)
**γ-herpesvirus- (MHV-68)**	–	–	–		7 (LB)		Cells & co-culture	CD10^neg^/EGFR^neg^	PCR	Infected HBCs secrete IL-1β which activates HUVECs to generate a pro-neutrophilic response	Hendrix et al. (92)
**SARS-CoV-2**	–	–	–		10 (LB)		Cells	CD10^neg^/EGFR^neg^	IF	No HBC infection with SARS-CoV-2	Lu-Culligan et al. (93)

HIV-1, Human immunodeficiency virus 1; NR, Not reported; RT, Reverse transcriptase; LB, Live birth; PCR, Polymerase chain reaction; IF, Immunofluorescence; ISH, In situ hybridization; PHA-PBMCs, Phytohemagglutinin stimulated peripheral blood cells; GM-CSF, Granulocyte-macrophage colony-stimulating factor; TNF, Tumor necrosis factor; IS-PCR, In situ PCR; MDMs, Monocyte-derived macrophages; DC-SIGN, Dendritic Cell-Specific Intercellular adhesion molecule-3-Grabbing Non-integrin; IL, Interleukin; TGF, Transforming growth factor; IFN, Interferon; VCCs, Virus containing compartments; FcγRI, Fc-gamma receptor 1; CMV, Cytomegalovirus; ZIKV, Zika virus; TOP, Termination of pregnancy; STB, Syncytiotrophoblast; GFP, Green fluorescent protein; HSV(-2), Herpes simplex virus (2); RSV, Respiratory syncytial virus; HUVECs, Human umbilical vein endothelial cells; SARS-CoV-2, sever acute respiratory syndrome coronavirus 2.

Permissiveness of HBCs depends on the HIV-1 strain utilized (63, 65, 68, 94). For example, Fear et al. (68) highlighted differences in viral replication among seven clinical and two laboratory-adapted strains, such as the macrophage-tropic HIV-Bal, and showed that only the laboratory-adapted strains and not the clinical isolates were able to replicate in HBCs.

Co-culture experiments of HBCs with different cell types *in vitro* have shown the importance of cell-cell interactions and cytokines in viral replication and hence a potential role of HBCs in viral dissemination. Viral replication in HIV-1-infected HBCs was enhanced after co-culture with CEM, a highly permissive T-cell line (62). Co-culture of infected syncytiotrophoblasts with non-infected HBCs increased HIV-1 gene expression in the former cells and secretion of IL6 and TNF-a by HBCs (70, 75).

Furthermore, intrinsic HBC factors could increase the risk of vertical HIV-1 transmission. The potential role of DC-SIGN in intrauterine transmission was discussed in the study of Boily-Larouche et al. (74), who detected the expression of DC-SIGN on HBCs derived from placentas of HIV-1-positive and -negative mothers and found that specific promoter variants enhanced the risk of vertical transmission. Reduced DC-SIGN expression was observed after promoter variant induction in HBCs from placentas of HIV-1-negative mothers and not those from HIV-1-exposed mothers (74). Decreased HIV-1 replication was observed when isolated HBCs from term placentas were infected with HIV-1 following small interfering ribonucleic acid (siRNA) knockdown of the proteinase inhibitor Cystatin B (CSTB). The levels of CSTB were lower in HBCs compared to MDMs isolated from seronegative donors, while viral protein was more abundant in MDMs (71). CSTB interacts with signal transducer and activator of transcription (STAT)-1 in both HBCs and MDMs, with the levels of STAT-1 tyrosine phosphorylation being higher in HBCs compared to MDMs and in uninfected HBCs compared to infected. Protein analysis of HBCs indicated an altered pattern of activation and signal transduction upon HIV-1 infection. Notably, the levels of STAT-1 serine phosphorylation didn’t alter upon infection and the different levels of phosphorylation suggest distinct regulation pathways (72). In addition, using primary HBCs from 10 term pregnancies, an increased susceptibility for HIV-1 and increased HIV-1 replication within HBCs was reported following CMV infection *in vitro* (77).

Finally, despite the ability of HBCs to replicate HIV-1, several factors can interrupt viral dissemination. Exogenous administration of IL-10 and TGF-β limited viral replication within HBCs while TNF-α pre-treatment significantly increased it (75). However, HBC co-culture with TNF-α or phytohemagglutinin (PHA)-stimulated peripheral blood mononuclear cells (PBMCs), which promotes the activation of T-cells, did not affect viral replication in HBCs (64). Of particular note, suppression of viral replication in HBCs was observed when treated with progesterone and zidovudine (67). HBCs were permissive to HIV-1 and formed virus-containing compartments (VCCs) enriched in tetraspanins, endosomal and lysosomal markers, and VCCs were accessible to neutralizing antibodies that could limit viral replication in HBCs through FcγRI (76).

#### ZIKV Observational Studies

In 2015-16, there was a Zika virus (ZIKV) epidemic, mainly affecting countries in South and Central America, as well as South East Asia, the Pacific Islands and parts of North America (95). During this epidemic, it was discovered that ZIKV can be transmitted vertically to the fetus, causing severe developmental complications including microcephaly (96, 97). This led to a number of studies that sought to study ZIKV infection in the placenta. Of these, nine studies investigated HBC infection and/or hyperplasia (increased HBC numbers) (**Table 1**) (39–47).

*In situ* analyses of placental tissue from ZIKV-infected pregnant women revealed ZIKV placental infection in 35% - 100% of samples (39–47) and HBC hyperplasia in all, except for one case-control study (45). In HBCs, ZIKV infection was assessed through detection of ZIKV protein (39, 42, 43, 45) or ZIKV ribonucleic acid (RNA) (40, 41) and both ZIKV RNA and protein were found in HBCs. No study investigated the presence of both ZIKV RNA and protein in HBCs nor reported the proportion of infected HBCs.

A range of gestational ages was studied, demonstrating the infection of HBCs in the first, second and third trimester. Most studies were small (n ≤ 3) (39–41, 43–46), but one larger study of 20 term placenta samples (42) suggested that ZIKV probably infects HBCs by term in all cases of maternal infection. A few studies reported the potential infection timepoint at which maternal infection occurred: first (39, 40, 42, 44–46), second (40, 42, 45) and third (39, 42, 45) pregnancy trimester.

Some studies reported fetal outcomes such as fetal infection or congenital malformations and HBC infection or/and hyperplasia (41, 43–47). Santos et al. (46) reported a case of placental inflammation, HBC hyperplasia and fetal infection. HBC hyperplasia in ZIKV-infected pregnancies has been observed in the presence (41) and absence (44) of placental inflammation and was correlated with fetal infection in both cases. Fetal defects were reported in cases with HBC hyperplasia (44), whereas no congenital anomalies were observed in the absence of HBC hyperplasia (45); a link between HBC hyperplasia and fetal defects could be suggested but needs further investigation. Increased numbers of HBCs expressing vascular permeability markers (vascular endothelial growth factor (VEGF)-2 and (C-C motif) ligands (CCL5) and pro-inflammatory cytokines (TNF-α and IFN-γ) were observed in immunohistochemistry experiments of a fetus with documented HBC infection and hyperplasia (43), suggesting a role of HBCs in the placental pro-inflammatory response, vascular permeability and a possible role of HBCs in transplacental transmission. Notably, fetal samples were not available for some studies and information on HBC infection/hyperplasia or fetal infection was often lacking.

#### ZIKV *In Vitro* Studies

Several groups have investigated ZIKV infection of HBCs *in vitro*, either by infecting isolated HBCs or placental explants (78–85) (**Table 2**). When isolated HBCs were infected *in vitro* with ZIKV, infection was identified through detection of viral protein (78–85) or/and viral RNA in HBCs (78, 79, 81, 83, 84), across all trimesters of pregnancy (78, 81, 84) (**Table 2**). However, different viral replication rates were observed among HBCs from different donors (81). An average percentage of 5% of isolated HBCs were infected (78, 81) and an average range of 10-37% in placental explants was documented (79, 82, 84). In addition, distinct infection rates were reported among different ZIKV strains in placental explants (82).

Some studies investigated the ability of ZIKV-infected HBCs and explants to produce mature virions capable of infecting other cells and thus contribute to vertical transmission. Production of virions by infected explants was observed in plaque assay experiments (82). HBCs were reported to replicate ZIKV that could infect epithelial Vero cells *in vitro* (79), and Janus kinase (JAK)-STAT signaling was important for the production of mature virions (83). Exposure of placental explants to ZIKV or/and flavivirus non-structural protein 1 (NS1) increased placental permeability, measured by dextran fluorescence permeability assay (85), suggesting a mechanism by which HBC-produced virions could potentially cross the placenta and transmit to the fetus.

HBCs express surface receptors that are important for ZIKV dissemination (80). The study of Tabata et al. (80) reported the expression of important surface viral entry cofactors T-cell immunoglobulin and mucin domain 1 (TIM1) and AXL tyrosine kinase receptors on HBCs. Co-cultivation of placental explants with duramycin, which prevents TIM1 receptor binding, reduced the viral load. However, changes in the expression of TIM1 or AXL in HBCs between infected and uninfected explants were not investigated (80).

Interestingly, a link between previously obtained immunity and ZIKV infection was recently described (84). The study of Zimmerman et al. (84) showed that pre-existing Dengue virus (DENV) antibodies enhanced ZIKV infection of HBCs *in vitro*, and the ZIKV-DENV complexes suppressed the production of antiviral effector proteins and retinoic acid-inducible gene-I-(RIG-I) like receptors, while treatment with IFN-β or blocking of FcRn binding reduced the viral replication rate (84). Regarding the presence of DENV cross-reactive antibodies *in vivo*, a few observational studies evaluated the presence of serum cross-reactive DENV antibodies with ZIKV antibodies but not with ZIKV (42, 43), while other studies reported previous DENV infection but did not check for the existence of cross-reactive antibodies (39).

ZIKV infection of HBCs and explants has been shown to alter HBC transcriptional and protein profiles (81, 83, 85). Infection of HBCs *in vitro* increased the transcription rate of several antiviral genes (IFNA, IFNB, IFIH1, DDX58, DHX58, IFIT1.2.3, OAS1, RSAD2), modestly upregulated surface proteins important for T-cell interactions (CD80, CD86 and MHC II) and elevated the secretion of cytokines/chemokines (IFN-α, IL-6, CCL2) and C-X-C motif chemokine ligand-10 (CXCL-10) (81). Of particular note, HBC activation was enhanced upon increased viral replication and, despite increased IFN-β mRNA, no IFN-β secretion was observed (81). Also, an increase in numbers of HBCs expressing DC-SIGN and HLA-DR proteins was reported (83). Downregulation of LYVE-1 - which regulates hyaluronic acid (HA) metabolism and induces HA-dependent leukocyte rolling - was observed (85). That decrease may imply an insufficient role of HBCs in maintaining vascular integrity of chorionic villi during infection and may therefore contribute to the alteration of placental permeability, as observed *in vitro* (85) and *in vivo* (43).

#### CMV Observational Studies

One of the most common transplacental infections in pregnancy, CMV, was examined in five observational studies (48–52) (**Table 1**). Absence of HBC infection was reported following immunohistochemistry analysis of placental tissue from CMV-infected women in two studies (48–50), while presence of viral DNA was observed (51, 52), highlighting differences in outcomes depending on the method used. Similarly to ZIKV infection studies, no study investigated the presence of both viral DNA and protein nor the percentage of infected HBCs. In addition, the trimester at which maternal infection or re-activation of infection occurred was not reported in any of the studies.

HBC hyperplasia was examined in two studies (49, 51), which found HBC hyperplasia in 100% of cases (n=7; **Table 1**) in the presence or absence of HBC infection. Euscher et al. (51) reported infection and hyperplasia of HBCs in three out of ten cases of unknown gestational ages. In contrast, Schwartz et al. (49) observed HBC hyperplasia in all four placentas from different gestational ages in absence of HBC infection.

Severe fetal or new-born outcomes were reported in the presence and absence of HBC infection (48–52). All examined cases of chorionic villitis with confirmed placental CMV infection (3 cases) exhibited fetal infection in the absence of HBC infection (48, 50). Also, three cases of lymphocytic villitis were examined and placental CMV inclusions were observed in two mothers and one reported fetal infection without identified viral positive HBCs (49). Euscher et al. (51) examined 10 cases of newborns with respiratory and neurological abnormalities and identified viral DNA in HBCs of 3 newborns, while Satosar et al. (52) examined 77 cases of Newborn mortality and morbidity and found four cases of CMV placental infection with CMV-positive HBCs.

#### CMV *In Vitro* Studies

Studies that have investigated HBC CMV infection and viral dissemination *in vitro* are shown in **Table 2**. Co-culture of isolated HBCs with CMV-infected syncytiotrophoblasts resulted in viral transmission to HBCs and induction of IL-8 and TGF-1β, whilst absence of the virus in co-culture experiments was reported after antibody-mediated blocking of cytokines, suggesting a role of those cytokines in viral gene expression that influences viral transmission (86). Wussow et al. (87) focused on the development of a vaccine based on modified vaccinia Ankara with CMV proteins and demonstrated that serum neutralizing antibodies against gH/gL were capable of preventing infection of HBCs, suggesting that pre-existing CMV antibodies could be captured by Fc receptors on HBCs and prevent viral spread by HBC-mediated neutralization.

Finally, changes in HBC immunophenotype during CMV infection were identified *in vitro* (77). Infection of HBCs with CMV led to upregulation of co-stimulatory (CD80) and immunoregulatory [programmed death-ligand 1 (PDL-1)] proteins and CCR5 co-receptor expression, increased the release of proinflammatory cytokines (TNF-α, IL-6), type I IFNs (IFN-α, IFN-β) and decreased the secretion of the IL-10 immunoregulatory protein (77). In addition, several antiviral gene transcripts were increased in HBCs, including type I IFNs and proteins contributing to type I IFN signaling (STAT-1, melanoma differentiation-associated protein-5 (MDA-5), RIG-I), while under-expression of STAT-2 and phosphorylated STAT-2 suggested involvement of CMV in transcription-translation interruption in HBCs (77).

#### Other Viruses

Apart from the effort to understand HBC response to HIV-1, ZIKV and CMV infection, the response of HBCs to other viral infections has been less extensively studied. Other viral infections examined included different types of herpes viruses (51, 52, 88, 89, 92), influenza (53, 54), parvovirus (51, 52), coxsackievirus (51), enterovirus (51), echovirus (89), Dengue virus (57), human papillomavirus (HPV) (56), hepatitis B (HBV) (55), respiratory syncytial virus (RSV) (91), Sendai virus (90), Chikungunya virus (58) and SARS-CoV-2 (25, 26, 59–61, 93).

#### Observational Studies of Other Viruses

Ten of thirteen observational studies investigated HBC infection by *in situ* detection of viral protein or/and viral DNA or RNA (**Table 1**). Both viral protein and RNA were detected in HBCs during coxsackievirus (51), enterovirus (52), H5N1 (54), and SARS-CoV-2 (26, 60) infection. Viral protein was also detected in HBCs following Dengue virus infection (57), viral RNA during Chikungunya virus infection (58), and viral DNA during herpes simplex virus types 1 and 2 (HSV) (51, 52) and HPV infection (56), while absence of parvovirus DNA (51, 52) and SARS-CoV-2 RNA and protein in HBCs (59, 61) was reported. Information regarding the timing of maternal infection during pregnancy was missing from all studies except the studies that investigated H5N1 infection, which occurred during the second pregnancy trimester and resulted in death (53, 54). The proportion of infected HBCs was only stated for coxsackievirus; reporting 10% positive cells for viral protein and 50% for viral RNA (51).

HBC hyperplasia was examined in eight observational studies. Coxsackievirus (51), parvovirus (51) and DENV infection (57) all induced HBC hyperplasia, whilst HBC hyperplasia was not seen during HSV infection (51). After maternal SARS-CoV-2 infection, no evidence of HBC hyperplasia was detected in one study (61), whilst three other studies identified HBC hyperplasia (25, 26, 59). For the other viral infections such as influenza, HBV, HPV and enterovirus, HBC hyperplasia was not examined.

Five of the observational studies looked at the presence of surface proteins including immunoglobulins on HBCs (25, 26, 53, 55, 60). HBCs express the human influenza receptor but not the avian receptor, with similar distribution between the infected and the control group, implying that distinct receptors or co-receptors are responsible for H5N1 entry in HBCs (53). Hepatitis B immunoglobulin (HBIG) was found on HBCs in placentas from HBV-positive mothers given intravenous HBIG (55). The proportion of HBIG was associated with fetal protection, potentially through viral neutralization by HBIG-HBCs (55). Expression of the checkpoint inhibitor PDL-1 was observed in HBCs following SARS-CoV-2 infection (26). Whether HBCs express the major SARS-CoV-2 entry receptor Angiotensin-converting enzyme 2 (ACE2) is still under debate, with one study reporting its absence in HBCs from 19 SARS-CoV-2-exposed placentas (25) and another reporting that ACE2 is expressed in the placenta, including HBCs, but downregulated upon SARS-CoV-2 infection (60).

Infection of HBCs has also been explored as a risk factor for transplacental infection or adverse pregnancy outcome. A large observational study involving 271 HPV-positive women identified placental infection in 31 placentas, with viral DNA present in HBCs, although HPV infection was not associated with pregnancy complications such as preterm birth or miscarriage (56). Fetal infection with DENV (57) and H5N1 (54) was documented and HBCs were infected in both cases. Placental analysis during DENV infection revealed increased deposition of proinflammatory cytokines (IFN-γ, TNF-α) and chemokines (CCL2, CCL5) in HBCs and vascular permeability factors such as VEGF2, VEGF receptor in the endothelium (57), proposing a barrier interruption and possible viral transmission through infected HBCs.

#### *In Vitro* Studies of Other Viruses

*In vitro* studies have shown that HBCs are permissive to MHV-68 (92), Sendai virus (90) and RSV (91) but not to HSV-2 (88, 89), echovirus 19 (89) or SARS-CoV-2 infection (93) (**Table 2**). The methods utilized include protein detection by immunofluorescence (88, 89, 91, 93), viral titer quantification (88–90), electron microscopy (89), polymerase chain reaction (PCR) (91, 92) and western blot (91). For *in vitro* studies of viral infection, HBCs were mostly isolated from term placentas, with only five out of thirty studies utilizing samples from the first or second trimester - the time period where congenital infection is most damaging.

The proportion of infected HBCs has only been reported for HSV (88) infection and RSV (91). Exposure to HSV resulted in infection of 17% of HBCs, measured by viral protein, whilst only 4.4% of cells produced infectious HSV, measured by viral titer assays (88). The proportion of RSV-infected HBCs varied between 5% to 17% among donors as measured by RFP fluorescence (91). This study also proposes a contribution of HBCs to RSV dissemination. Although no detectable RSV was observed in HBC supernatant 30 days after *in vitro* infection, co-culture of infected HBCs with human bronchial epithelial cells spread the virus, suggesting a vital role of cell-to-cell interaction for viral dissemination.

Interestingly, HBCs were competent to eliminate HSV-2 and echovirus 19 infection (89). During those infections, pseudopods were emitted from HBCs, and there were increases in lipid accumulation, cellular volume, and numbers of vacuoles in cytoplasm, suggesting viral phagocytosis and degradation within HBCs (89).

An altered secretion profile has been observed upon viral infection of HBCs *in vitro*. Proinflammatory cytokines were released from HBCs during MHV-68 (IL-1β) (92), Sendai virus (IFN-β) (90) and RSV (TNF-α, IFN-γ, IL-6, IL-12) (91) infection. Interestingly, the IL-1β secretion during MHV-68 infection induced IL-8 secretion and increased pro-neutrophil response marker expression (IL-8, vascular cell adhesion molecule-1, intercellular cell adhesion molecule-1 (ICAM-1), E-selectin) in human umbilical vein endothelial cells (HUVECs) (92). This indicates a possible role of HBCs in creation or maintenance of a proinflammatory environment and activation of other cells in the placenta to counter pathogen invasion.

### HBCs in Bacterial Infection

#### Observational Studies of Bacterial Infection

Some bacterial infections are associated with pregnancy complications followed by adverse pregnancy outcomes (98) (**Table 3**). The used search strategy obtained two observational studies which examined HBC bacterial infection or hyperplasia (52, 103). An increase in the number of HBCs was reported in *Treponema pallidum*-infected placentas from mothers with placental villitis (103). From a total of 15 cases of offspring death or illness in which placental bacterial infection was identified (using a consensus probe), mainly trophoblast rather than HBC infection was reported, in contrast to viral infection (52). The gestational age of maternal infection in pregnancy was not reported for any of these 21 cases of bacterial infection (52, 103).

**Table 3:** Hofbauer cells in observational studies of parasitic and bacterial infection.

Pathogen	Gestation of samples (outcome)		Trimester of infection	Diagnosis of HBC infection	HBC infection (no.)	HBC hyperplasia (no.)	Key outcome(s) in HBCs	Reference
	Preterm	Term						
***Plasmodium falciparum***	9 (NR)	14 (NR)	NR	NR	NR	NR	Similar CCR5 expression in HBCs from infected and uninfected women	Tkachuk et al. (99)
	–	9 (LB)	NR	NR	NR	NR	No significant difference in HBCs MIF expression between PM+ and PM- placentas	Chaisavaneeyakorn et al. (100)
	3 (LB)	14 (LB)	NR	Hemozoin detection	+ (NR)	NR	Hyperplasia of HBCs and decrease in M2 percentage associated with low-birth weight in first pregnancies	Gaw et al. (101)
	33 (CA)	8 (LB) & 26 (CA)	NR	NR	NR	NR	Identification of IgE positive HBCs	Rindsjö et al. (102)
***Treponema pallidum***	2 (1 LB & 1 PL)	4 (3 LB & 1 PL)	NR	NR	NR	+ (100%)	Hyperplasia of HBCs was observed in *Treponema pallidum* infected placentas	Walter et al. (103)
**Various bacteria, including *E. coli, Klebsiella, Streptococcus agalactiae***	15, gestational age NR (PL and LB; numbers NR)		NR	rRNA sequence	- (NR)	NR	Mainly trophoblast rather than HBC infection was identified	Satosar et al. (52)
***E.coli*, *Haemophilus influenza*, GBS (CA patients)**	11 (LB) & 9 (CA)	–	NR	NR	NR	NR	Differences in HBC biology among healthy individuals and CA patients	Amara et al. (104)

NR, Not reported; CCR5, C-C chemokine receptor type 5; LB, Live birth; MIF, Migration inhibitory factor; PM, Placental malaria; PL, Pregnancy loss (stillbirth/miscarriage); E. coli, Escherichia coli; GBS, Group B Streptococcus; CA, Chorioamnionitis.

#### *In Vitro* Studies of Bacterial Infection

Several groups have investigated HBC responses to bacterial infection *in vitro*, including 5 studies of GBS (104–108), *Coxiella burnetii* (109)*, E. coli* (104, 105) and *Lactobacillus crispatus* and *E.coli* infections (110). Most of the studies used third trimester placentas. The sample size and gestational age were often not reported (**Table 4**).

**Table 4:** Hofbauer cells in *in vitro* studies of bacterial infection.

Bacteria	Samples studied by trimester (outcome)				Model(s) of infection	Immuno-purification of HBCs	Diagnosis of HBC infection/ viral replication (no.)	Key outcome(s) in HBCs	Reference
	1^st^	2^nd^	3^rd^ <37w	3^rd^ ≥37w					
**Unopsonized GAS bioparticles**	–	–	–	3-5 (LB)	Cells	CD14^pos^	Fluorometric phagocytosis assay	PGE2 regulates HBC phagocytic ability	Mason et al. (107)
**Unopsonized GBS & *E.coli* bioparticles**	–	–	–	3 (LB)	Cells	CD14^pos^	Fluorometric phagocytosis assay	PGE2 regulates HBC phagocytic ability	Rogers et al. (108)
**GBS & *E.coli***	–	9 CA (LB)	11 (LB)	–	Cells	CD14^pos^	NR	HBCs from CA patients are altered compared to HBC from normal pregnancies	Amara et al. (104)
**GBS, *E.coli* & killed bacteria**	–	–	–	3 (LB)	*Ex-vivo &* cells	CD14^pos^	Microscopy (NR)	HBCs release METs with digestion enzymes upon GBS and *E.coli* infection	Doster et al. (105)
**GBS**	NR				Cells	CD14^pos^	Colony forming units (NR)	HBC immune response to GBS is dependent on protein kinase D	Sutton et al. (106)
***Coxiella burnetii***	–	–	46 (LB)		Cells	CD14^pos^	PCR & microscopy (NR)	HBCs eliminate *Coxiella burnetii* through IFN-γ	Mezouar et al. (109)
***Lactobacillus crispatus, E.coli* and labeled *E.coli***	20 (TOP)	–	–	–	Cells	Multiparameter flow cytometry	Colony forming units (NR)	HBCs eliminate *Lactobacillus crispatus* and *E.coli*, and have phagocytic capacity	Thomas et al. (110)

GAS, Group A Streptococcus; LB, Live birth; PGE2, Prostaglandin E2; GBS, Group B Streptococcus; E. coli, Escherichia coli; NR, Not reported; CA, Chorioamnionitis; METs, Macrophage extracellular traps; PCR, Polymerase chain reaction; IFN, Interferon; TOP, Termination of pregnancy.

Alterations in HBC numbers and phenotype have been reported in cases of chorioamnionitis (104). Amara et al. (104) examined nine patients with chorioamnionitis (4 grade I, 3 grade II and 2 grade III), of whom two had vaginal GBS and one had vaginal *E.coli*, three had bacteriuria and two had *E.coli* in their placentas. The numbers of HBCs were remarkably reduced in chorioamnionitis cases compared to HBCs from normal pregnancies and the expression of CD163, monocyte/macrophage scavenger receptor shed by inflammatory stimuli, was significantly increased in severe compared to moderate cases. CD163 expression was also increased in HBCs from chorioamnionitis-affected placentas with documented infections compared to those without an identified infection, suggesting this is a response to infection rather than sterile inflammation. The expression of M2 and M1 macrophage phenotype markers was similar between chorioamnionitis-positive and -negative pregnancies, and only three M1 genes (EDN1, IL-15 and IL-15RA) were reported as upregulated in HBCs from chorioamnionitis-positive cases (104).

HBCs alter their inflammatory profile to cope with *in vitro* bacterial infection. GBS infection of HBCs shifts their immunophenotype by increasing the secretion of proinflammatory cytokines (IL-6, IL-1β and TNF-α), activation of the nuclear factor kappa B (NFκβ), inflammasome assembly and upregulation of E-Selectin and the chemokine CCL4 in a protein kinase D-dependent manner (106). HBCs infected with *Coxiella burnetii* upregulate eight M1 macrophage-associated genes, whilst increased release of IFN-γ was associated with bacterial elimination (109).

In addition to the ability of HBCs to eliminate pathogen infection by cytokine induction, Doster et al. (105) proposed a novel mechanism of bacterial elimination by the release of macrophage extracellular traps (METs). Upon GBS infection, HBCs were observed to release METs containing matrix metalloproteases capable of killing infected cells. The release of METs is not GBS-specific, with other bacteria such as *E. coli* and heat-killed GBS bacteria leading to similar MET release levels, suggesting a broad defense mechanism. Interestingly, MET release may contribute to fetal membrane weakening since METs which contained matrix metalloproteases and CD163-positive cells were found on fetal membranes (105).

Finally, three studies reported the phagocytic capacity of HBCs using labelled bacteria (110) and unopsonized bacterial bioparticles (107, 108). Using unopsonized bioparticles, it has been shown that prostaglandin E2 (PGE2) regulates the phagocytic ability of HBCs to take up Group A *Streptococcus* (107), GBS and *E. coli* (108).

### HBCs in Parasitic Infection

Only four observational studies of HBC responses to parasitic infections were identified, and all of them focused on *Plasmodium falciparum* (99–102) (**Table 3**). One of these investigated HBC infection indirectly, by identifying parasite disposal products in HBCs (101), one investigated the presence of IgE in association with HBCs (102), and the other two investigated HBC protein expression. Similar expression of the macrophage migration inhibitory factor (MIF) (100) and HIV-1 co-receptor CCR5 (99) was reported in HBCs between infected and uninfected women. A possible association between the HBC inflammatory response and fetal growth restriction in malaria-infected women was reported (101). Specifically, an increase in HBC numbers was observed and analysis of the CD163:CD68 balance in HBCs revealed a decrease in the M2 phenotype, which was associated with lower birth weight (101).

### HBC Responses to Pathogen Associated Molecular Patterns (PAMPs)

As an alternative to studying responses to whole pathogens, several groups have investigated HBC responses to individual pathogen-associated molecular patterns (PAMPs) including lipopolysaccharide (LPS) (6, 69, 104, 109–115), merozoite surface protein 1 (MSP1) (116), polyinosinic:polycytidylic acid (PIC) and peptidoglycan (PGN) (110, 114), and Imiquimod and FSL-1 stimulation (110) (**Table 5**). HBCs were isolated mainly from third trimester placentas (n=9 studies) (6, 69, 109, 111–116), with only three studies using HBCs from the first or second trimester (6, 110, 112).

**Table 5:** Studies that investigated HBC response to PAMP(s) treatment *in vitro.*.

Treatment	Samples studied by trimester (outcome)				Model(s) of infection	Immuno purification of HBCs	Key outcome(s) in HBCs	Reference
	1^st^	2^nd^	3^rd^ <37w	3^rd^ ≥37w				
**LPS**	–	–	–	NR (LB)	Cells	No	HBCs secrete lower levels of cytokines compared to MDMs and LPS-treated HBCs	Plaud-Valentin et al. (69)
	–	–	–	12 (LB)	Cells	No	LPS treatment increased the percentage of cytokine producing HBCs	Pavlov et al. (111)
	–	9 CA (LB)	11 (LB)	–	Cells	CD14^pos^	Differences in inflammatory response of HBCs, MGCs and HBCs from CA patients	Amara et al. (104)
	13-24 (TOP)	–	–	13-24 (LB)	Cells	No	LPS treatment increased cytokine release by HBCs	Pavlov et al. (112)
	–	–	46 (LB)		Cells	CD14^pos^	LPS did not induce M1-like transcriptional profile in HBCs	Mezouar et al. (109)
**LPS & IFN-γ**	8 (TOP)		–	5 (LB)	Cells	CD14^pos^	Difference in HBC response to LPS/IFN-γ treatment from early/midgestational and term pregnancies	Swieboda et al. (6)
	–	–	–	5 (LB)	Cells	CD10^neg^/EGFR^neg^	HBCs maintain their M2 phenotype despite treatment with LPS/IFN-γ and alter their cytokine profile	Schliefsteiner et al. (113)
**LPS, PIC & PGN**		–	–	16 (LB)	Cells	CD10^neg^/EGFR^neg^	HBC inflammatory responses can be induced despite their M2 phenotype	Young et al. (114)
**MSP1**	–	–	–	6 (LB)	Perfusion model	No	No immune complexes identified in HBCs	May et al. (116)
**LPS & ATP**	–	–	–	6 (LB)	Cells	CD10^neg^/EGFR^neg^	Inflammasome activation in HBCs, release of IL-1β and pyroptotic cell death	Abrahams et al. (115)
**LPS, LPS & IFN-γ, PIC, Imiquimod, PGN, FSL-1**	20 (TOP)	–	–	–	–	Multiparameter flow cytometry	HBCs respond to toll-like receptor agonist stimulation Among the stimulants, FSL-1 and LPS & IFN-γ have the greatest impact FSL-1 enhanced the production of CCL-3, IL-8, IL-6, and GM-CSF LPS & IFN-γ enhanced the secretion of TNF-α, IL-1β and reduced the production of TIMP-1 and MMP-9	Thomas et al. (110)

LPS, Lipopolysaccharides; NR, Not reported; LB, Live birth; MDMs, Monocyte-derived macrophages; CA, Chorioamnionitis; MGCs Multinucleated giant cells; TOP, Termination of pregnancy; IFN, Interferon; PIC, Polyinosinic,polycytidylic acid; PGN, Peptidoglycan; MSP1, Merozoite surface protein 1; ATP, Adenosine triphosphate; IL, Interleukin; FSL-1, Pam2CGDPKHPKSF-synthetic lipopeptide; CCL, C-C motif chemokine ligand; GM-CSF, Granulocyte-macrophage colony-stimulating factor; TNF, Tumor necrosis factor; TIMP-1, TIMP metallopeptidase inhibitor 1; MMP-9, Matrix metallopeptidase 9.

Lipopolysaccharide (LPS) was the most commonly used bacterial PAMP in HBC studies, LPS is a membrane component of gram-negative bacteria that signals mainly through toll-like receptor 4 (TLR4). One recent study performed an analysis of HBC response to LPS across gestation (6), and found a significant amount of activated HBCs expressing tolerogenic markers in early pregnancy stages, with the number of activated cells dropping at term. Treatment with IFN-γ and LPS led to the expression of inducible nitric oxide synthase (iNOS) and STAT-1 by early/midgestational HBCs, while the M1 to M2 phenotype ratio was not affected in term HBCs. More specifically, several subtypes of M2 phenotypes were detected at term, suggesting that HBCs are a heterogenous population of macrophages with regulatory and anti-inflammatory properties orchestrated in time through pregnancy (6). Thomas et al. (110) also showed that LPS and IFN-γ treatment enhanced the secretion of inflammatory cytokines (TNF-α and IL-1β) and reduced the production of tissue remodeling factors (TIMP-1 and MMP-9), while FSL-1 (TLR2/TLR6 agonist) treatment enhanced the production of chemoattractants and cytokines (CCL-3, IL-8, IL-6, and GM-CSF) in 3^rd^ trimester.

Mezouar et al. (109) proposed that HBCs from term placentas maintain an M2 anti-inflammatory phenotype in the presence of LPS, despite the induction of M1-like proteins and cytokines, unlike *in vitro* bacterial infections. Schliefsteiner et al. (113) examined the HBC response in the presence of LPS and IFN-γ, which did not induce M1 polarization but a decrease in the percentage of HBCs expressing M2 type associated proteins (CD206 and DC-SIGN). In addition, they observed an elevated release of proinflammatory cytokines (TNF-α and IL-12) but also release of M2-immunoregulatory (IL10), highlighting the heterogenicity of HBCs and the oversimplified classification of macrophage phenotypes (113). Plaud-Valentin et al. (69) also observed the release of TNF-α and IL-1 from IFN-γ and LPS-stimulated HBCs; however, the cytokine secretion was higher in monocyte-derived macrophages. Similarly, an increase in the percentage of HBCs with intracellular expression of proinflammatory cytokines IL-1α, IL-1β, TNF-α and IL-6 was reported upon LPS stimulation by Pavlov et al. (111). Inflammasome activation, increased release of IL-1β and pyroptosis were induced upon HBC treatment with LPS and ATP (115). Robust secretion and transcription of the IL-6 pro-inflammatory cytokine and to a smaller degree of IL-8 chemokine was documented upon HBC treatment with LPS and to a lesser extent with PIC treatment, while PGN did not affect IL-6 and IL-8 secretion (114).

LPS responses by HBCs from normal pregnancy and those with chorioamnionitis were compared by Amara et al. (104). HBCs were shown to release IL-6, IL-10 and TNF-α in response to LPS. This study also observed, for the first time, the spontaneous *in vitro* formation of multinuclear giant cells (MGCs) from HBCs in healthy pregnancies. These MGCs exhibited similar phagocytic capacity to HBCs and released smaller amounts of TNF-α in response to LPS (though similar levels of IL-6 and IL-10). Interestingly, in cases of chorioamnionitits, they saw reduced HBC numbers, potentially *via* increased apoptosis or recruitment of HBCs into inflamed fetal tissues, as well as a failure by the cells to form MGCs. LPS treatment of HBCs from chorioamnionitis patients demonstrated reduced IL-10 responses and defective activation of the signal transducer and activator of transcription 1 (STAT-1), which is important for LPS-stimulated gene expression.

## Discussion

**Figure 3:**
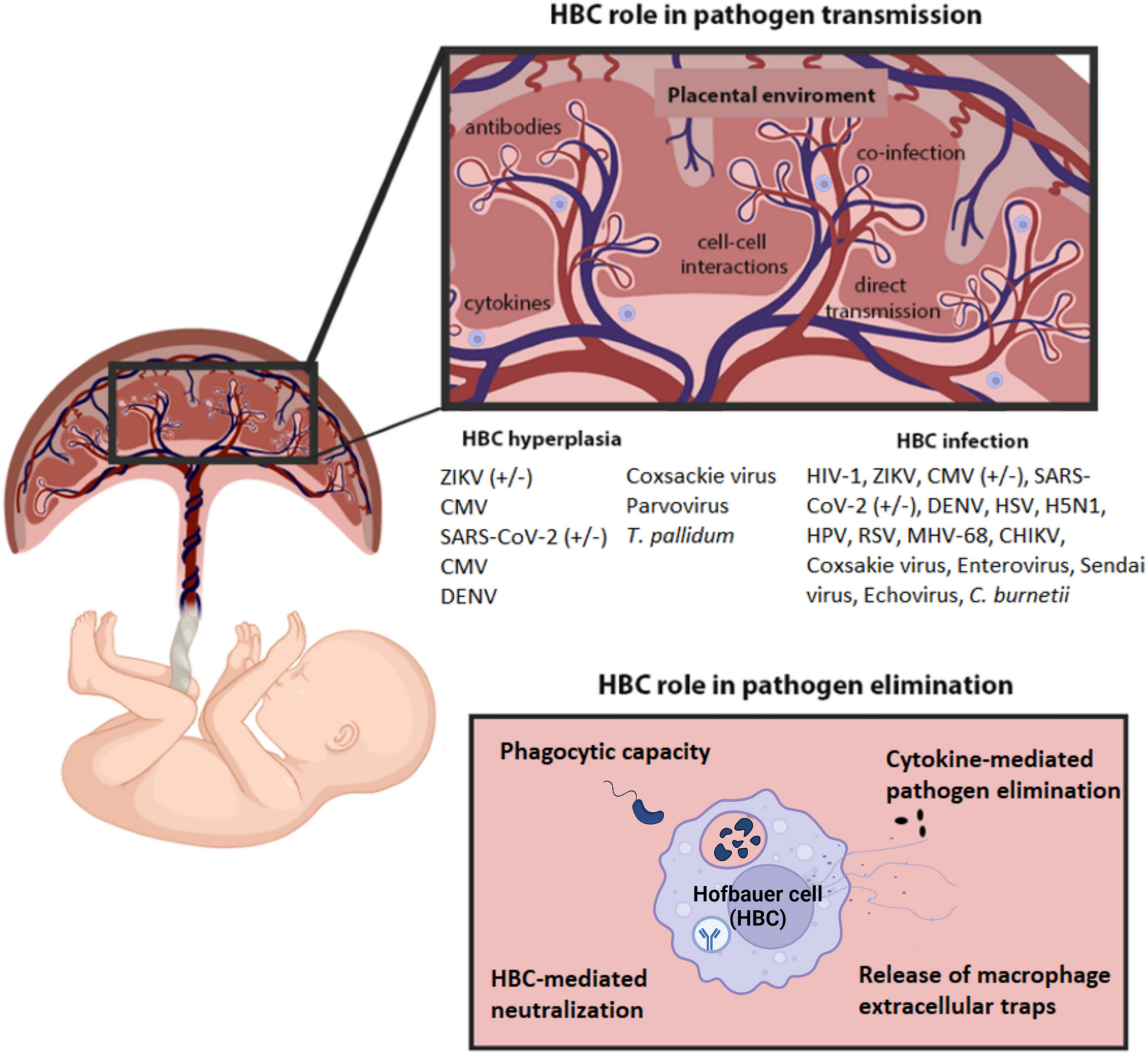
Overview of the role of HBCs in pathogen infection and pathogen elimination. HBCs can be infected by a range of pathogens, which in some cases can replicate and transmit to other cells. Some pathogens induce HBC hyperplasia which may contribute to pathogen spread by increasing pathogen reservoirs. Release of cytokines during infection may contribute to pathogen elimination or enhance pathogen replication in HBCs. Other factors, such as antibodies and cytokines also influence HBC susceptibility to infection. However, HBCs also have the capacity to eliminate pathogen invasion by phagocytosis or the release of METs and pathogen neutralization. Created with BioRender.com.

**Figure 4:**
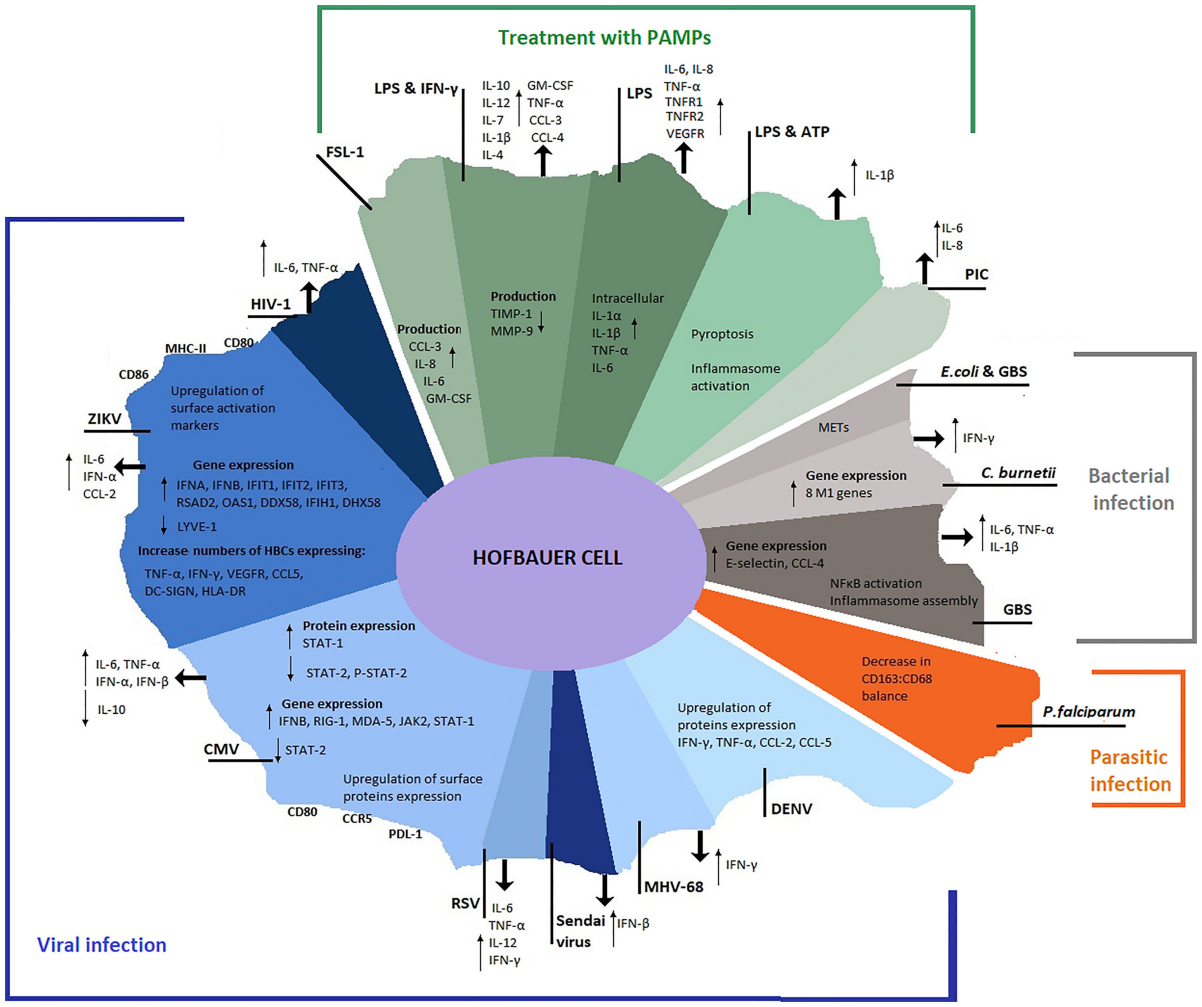
Overview of HBC profile during pathogen infection and PAMP(s) stimulation. Alterations in HBC transcriptional, translational and secretional levels documented after pathogen infection or PAMP(s) treatment in the studies retrieved from the search strategy. Viral infections are represented in blue shades, parasitic infections in orange, bacterial infections in grey and PAMP(s) treatments in green.

Here we present a systematic scoping review of the role of Hofbauer cells in infection during pregnancy. A variety of pathogens productively infect, invade, or modulate HBCs as revealed using *in situ*, *ex vivo* and *in vitro* approaches (Summarized in **Figures 3** and **4**). HBCs can exert responses that are highly infection-restrictive and protective to the fetus (87, 92, 105, 110, 115), but can also be exploited to the advantage of the pathogen for dissemination or hiding (76, 91, 113). Therefore, HBCs appear to be a “double-edged sword” in the context of placental and congenital infection. Several studies described the presence of HBC hyperplasia during infection in the placenta, supporting their role in fetal defense against invading pathogens (25, 26, 39–44, 46, 47, 49, 51, 57, 59, 103). It is not known whether this is a result of replication of existing HBCs in the villi or through migration of additional cells from the fetus. Only one study demonstrated an increase in HBC proliferation upon infection- a study of ZIKV infection (82), hence further studies are needed to determine the potential role of HBC proliferation during infection by other pathogens.

Different pathogens and PAMPs uniquely influence HBC responses inducing alterations in their transcriptional, translational and secretion profiles. Although HBCs have been defined as M2-like macrophages that maintain an anti-inflammatory phenotype, characterized by contributions to tissue maintenance and developmental regulation (4, 5), pathogens or PAMPs have been reported to induce potent M1-like responses in these cells (109), with HBCs mounting robust pro-inflammatory cytokine responses  (92, 114, 115). Conversely, HBCs have also been reported to maintain an M2-like phenotype during pathogen encounter  (109, 113). A number of pro-inflammatory cytokines are released following exposure to viral and bacterial infections and upon PAMP exposure (**Figure 4**). These reported alterations in HBC profile during infection suggest that HBCs have evolved mechanisms to enable their activation and stimulation of adaptive immune responses through antigen presentation, however secretion of some cytokines could also aid in virus dissemination (70).

Of the clinically relevant viruses that are known to infect the fetus *in utero*, HIV, ZIKV and CMV have been proven as capable of establishing infection in the placenta (34, 78, 86) but specific interactions with HBCs are still far from fully characterized. Infection by HIV, for example, has been shown to occur *in vitro*, though clinical strains were inferior at infecting HBCs compared to laboratory-adapted strains (65, 66, 68), and HBCs were more resistant to production of new viral progeny than monocytes or monocyte-derived macrophages (MDMs) (71, 73), suggesting that more studies are needed to establish how efficiently HIV can replicate within HBCs. Even though the field is more recent, ZIKV infection of HBCs has been more extensively studied following the establishment of its link to congenital abnormalities such as microcephaly, and HBCs could therefore be important for ZIKV cross-placental transmission. However, it is not completely understood whether it is through productive replication or migration and cell-to-cell spread that HBCs contribute to dissemination towards the fetal compartment. Hyperplasia during placental ZIKV infection has been observed in a number of studies (39, 40, 42–44, 46, 47).

Despite being the most common congenital virus, CMV is not well characterized in terms of its ability to infect HBCs. *In situ* studies involve only a few samples (48–52) and the number of *in vitro* studies is small (77, 86, 87). Thus, it is not clear whether HBCs are routinely infected during placental infection by CMV. Furthermore, additional *in vitro* investigations are necessary to understand how HBCs interact with and respond to CMV and what implications that may have for preventing congenital infection. It would be of interest to explore what other viruses may escape “under the radar” during pregnancy, particularly where overt disease is not seen in the neonate. Multiple studies indicate other viruses such as HSV and coxsackie virus may also be clinically relevant and should be investigated further in the context of placental and HBC infection (51, 52). Additional studies describe capability of other viruses to infect and replicate within HBCs (26, 51, 52, 54, 56–58, 60, 90–92), though it is not known whether these also possess the ability to be systemically spread to reach the placenta and the fetal compartment due to their specific tropism profiles.

An inflammatory microenvironment induced by PAMPs, pathogens and a broader alteration of placental microenvironment may influence the profile of HBCs without direct infection (104). Most of the studies that documented placental and HBC infection identified pathogen genome or antigens within HBCs; however, identifying pathogen components does not reflect live infection. Extracellular vesicles could transfer pathogen components from infected cells to other cell types (117); however, this communication mechanism has not been investigated in HBCs. In this case, plaque assays or culture experiments could reveal the presence of live virus or bacteria, respectively, and thus reflect the capacity of HBCs to replicate pathogens. Infection-like microenvironments induced by PAMPs compared to damage-associated molecular patterns induce different HBC proliferation rates (118) and potentially different HBC expression patterns. Furthermore, supraphysiological doses of individual PAMPs used in *in vitro* experiments do not recapitulate real infection, and there are documented differences in HBC profiles between pathogen infection and PAMP treatment (109) (Summarized in **Figure 4**).

Finally, HBCs reside in several micro-locations within the chorionic villus (119), but how these spatial differences affect permissiveness to pathogens and immune functions remains to be investigated. Specifically, HBCs within the anchoring villi are closer to the decidua than those within the floating villi. Since some pathogens are thought to infect the placenta by invading extravillous trophoblasts in the anchoring villi (14), the phenotype and the proportion of infected HBCs within anchoring villi may be different compared to HBCs in floating villi. Also, the influence of decidual cells, including immune cells, on HBC behavior could be higher in anchoring villi, which would have higher exposure to secreted factors. Current investigations of *in vitro* HBC responses have not examined the effect of HBC location on their response to pathogens and likelihood of infection; this could be beneficial to understanding their role in protecting the fetus from congenital infections.

In completing this review, we identified some themes, including strengths and weaknesses of existing studies, the significance of HBC isolation technique in interpreting findings, the importance of considering pathogen characteristics when designing these studies, which we have summarized in **Table 6**. Collectively, elucidating the nature of interactions between HBCs and placental pathogens is of vital importance and relevance to understanding the infection-induced mechanisms leading to adverse pregnancy outcomes.

**Table 6:** Considerations for future microbial studies in placental macrophages.

Minimal reporting for clinical samples	Considerations when studying isolated HBCs
Pregnancy outcome (e.g. termination, pregnancy loss or live birth, gestation at delivery)	Isolation procedure: using initial trypsin digests of placenta will result in contamination with maternal macrophages
Presence/absence of fetal/newborn infection	Isolation procedure: depletion of non-macrophage cells by immunomagnetic isolation should be performed
Presence/absence of chorioamnionitis	
Fetal sex	Isolation procedure: positive immunoselection of CD14+ macrophages will cause cell activation
The gestation of pregnancy at maternal infection	
Method used for infection diagnosis	*In vitro* direct exposure to high-dose pathogens does not recapitulate how HBCs encounter pathogens *in vivo*
The number of patients studied	
Sampling methodology for placental biopsies	HBC permissiveness to pathogens could be affected by monoculture versus co-culture with other placental cells
Gestation of placental samples	
The proportion of HBCs with evidence of infection	
**Considerations for *in vitro* pathogen challenge**	**Unanswered/less explored research questions**
The strain of virus or bacteria is important – macrophage tropism and clinical strains should be considered	To what extent HBCs alter their response, susceptibility, and permissiveness to infection throughout gestation
Ability for viruses to enter cells should be clearly distinguished from ability to replicate	Whether past infection history influences infection susceptibility/transmission during pregnancy
Techniques that measure viral infection using protein and nucleic acid should be complemented with techniques that measure live infectious virus	To what extent HBC localisation (both within villi and across the placenta) alter their phenotype and function
	The role of fetal sex in HBC responses to infection
Possibility of replication followed by entrapment inside cells should be considered and experimentally tested.	Few studies have investigated first/second trimester HBC responses to pathogens *in vitro*
Rate of infection – the use of high multiplicity of infections (MOI) may affect outcomes	In what way inflammation in response to infection may be beneficial in preventing spread to the fetus

## Data Availability Statement

The original contributions presented in the study are included in the article/**Supplementary Material**. Further inquiries can be directed to the corresponding author.

## Author Contributions

BH conceived the review. GF led the screening of the papers, with assistance from PP and BH. GF drafted the review with BH, PP, and VB contributing to the writing and editing. All authors contributed to the article and approved the submitted version.

## Funding

Research reported in this publication was supported by the Eunice Kennedy Shriver National Institute of Child Health and Human Development, of the National Institutes of Health under award number 5R01HD093801.

## Author Disclaimer

The content is solely the responsibility of the authors and does not necessarily represent the official views of the National Institutes of Health.

## Conflict of Interest

The authors declare that the research was conducted in the absence of any commercial or financial relationships that could be construed as a potential conflict of interest.

## Publisher’s Note

All claims expressed in this article are solely those of the authors and do not necessarily represent those of their affiliated organizations, or those of the publisher, the editors and the reviewers. Any product that may be evaluated in this article, or claim that may be made by its manufacturer, is not guaranteed or endorsed by the publisher.

## References

[1] Castellucci M Zaccheo D Pescetto G . A Three-Dimensional Study of the Normal Human Placental Villous Core - I. Hofbauer Cells Cell Tissue Res (1980) 210(2):235–47. doi: 10.1007/BF00237612 7407868

[2] Loegl J Hiden U Nussbaumer E Schliefsteiner C Cvitic S Lang I . Hofbauer Cells of M2a, M2b and M2c Polarisation May Regulate Feto-Placental Angiogenesis. Reproduction (2016) 152(5):447–55. doi: 10.1530/REP-16-0159 27534571

[3] Seval Y Korgun ET Demir R . Hofbauer Cells in Early Human Placenta: Possible Implications in Vasculogenesis and Angiogenesis. Placenta (2007) 28(8–9):841–5. doi: 10.1016/j.placenta.2007.01.010 17350092

[4] Khan S Katabuchi H Araki M Nishimura R Okamura H . Human Villous Macrophage-Conditioned Media Enhance Human Trophoblast Growth and Differentiation *In Vitro* . Biol Reprod (2000) 62(4):1075–83. doi: 10.1095/biolreprod62.4.1075 10727280

[5] Anteby EY Natanson-Yaron S Greenfield C Goldman-Wohl D Haimov-Kochman R Holzer H . Human Placental Hofbauer Cells Express Sprouty Proteins: A Possible Modulating Mechanism of Villous Branching. Placenta (2005) 26(6):476–83. doi: 10.1016/j.placenta.2004.08.008 15950061

[6] Swieboda D Johnson EL Beaver J Haddad L Enninga EAL Hathcock M . Baby’s First Macrophage: Temporal Regulation of Hofbauer Cell Phenotype Influences Ligand-Mediated Innate Immune Responses Across Gestation. J Immunol (2020) 204(9):2380–91. doi: 10.4049/jimmunol.1901185 PMC7870092 32213562

[7] Goldstein J Braverman M Salafia C Buckley P . The Phenotype of Human Placental Macrophages and Its Variation With Gestational Age. Am J Pathol (1988) 133(3):648–59. PMC1880826 3264459

[8] Mues B Langer D Zwadlo G Sorg C . Phenotypic Characterization of Macrophages in Human Term Placenta. Immunology (1989) 67(3):303–7. PMC1385344 2788125

[9] Mosser DM Edwards JP . Nihms84393. Nat Rev Immunol (2009) 8(12):958–69. doi: 10.1038/nri2448 PMC2724991 19029990

[10] Zulu MZ Martinez FO Gordon S Gray CM . The Elusive Role of Placental Macrophages: The Hofbauer Cell. J Innate Immun (2019) 11:447–56. doi: 10.1159/000497416 PMC6758944 30970346

[11] WHO . Congenital Anomalies [Internet] (2020). Available at: https://www.who.int/news-room/fact-sheets/detail/congenital-anomalies.

[12] Torgerson PR Mastroiacovo P . The Global Burden of Congenital Toxoplasmosis: A Systematic Review. Bull World Health Organ (2013) 91(7):501–8. doi: 10.2471/BLT.12.111732 PMC3699792 23825877

[13] WHO . The Global Elimination of Congenital Syphilis: Rationale and Strategy for Action [Internet]. Geneva: WHO Press (2007). Available at: https://apps.who.int/iris/bitstream/handle/10665/43782/9789241595858_eng.pdf?sequence=1&isAllowed=y.

[14] Neu N Duchon J Zachariah P . TORCH Infections. Clin Perinatol (2015) 42(1):77–103. doi: 10.1016/j.clp.2014.11.001 25677998

[15] Arora N Sadovsky Y Dermody TS Coyne CB . Microbial Vertical Transmission During Human Pregnancy. Cell Host Microbe (2017) 21(5):561–7. doi: 10.1016/j.chom.2017.04.007 PMC6148370 28494237

[16] Boyle AK Rinaldi SF Norman JE Stock SJ . Preterm Birth: Inflammation, Fetal Injury and Treatment Strategies. J Reprod Immunol (2017) 119:62–6. doi: 10.1016/j.jri.2016.11.008 28122664

[17] Frenkel DL . Infectious Diseases as a Cause of Global Childhood Mortality and Morbidity: Progress in Recognition, Prevention, and Treatment. Adv Pediatr Res (2018) 5(14):1–11. doi: 10.24105/apr.2018.5.14

[18] Shmueli E Hadar E Pardo J Attias J Amir J Bilavsky E . Congenital Cytomegalovirus Infection After a Multiple Birth Pregnancy. Pediatr Infect Dis J (2017) 36(12):e298–302. doi: 10.1097/INF.0000000000001725 28763424

[19] Melo AS Aguiar RS Amorim MMR Arruda MB De Oliveira Melo F Ribeiro STC . Congenital Zika Virus Infection: Beyond Neonatal Microcephaly. JAMA Neurol (2016) 73(12):1407–16. doi: 10.1001/jamaneurol.2016.3720 27695855

[20] Kovo M Ganer Herman H Gold E Bar J Schreiber L . Villitis of Unknown Etiology – Prevalence and Clinical Associations. J Matern Neonatal Med (2016) 29(19):3110–4. doi: 10.3109/14767058.2015.1114090 26512448

[21] Tita ATN Andrews WW . Diagnosis and Management of Clinical Chorioamnionitis. Clin Perinatol (2010) 37(2):339–54. doi: 10.1016/j.clp.2010.02.003 PMC3008318 20569811

[22] Menter T Mertz KD Jiang S Chen H Monod C Tzankov A . Placental Pathology Findings During and After SARS-CoV-2 Infection: Features of Villitis and Malperfusion. Pathobiology (2021) 88(1):69–77. doi: 10.1159/000511324 32950981 PMC7573905

[23] Cribiù FM Erra R Pugni L Rubio-Perez C Alonso L Simonetti S . Severe SARS-CoV-2 Placenta Infection can Impact Neonatal Outcome in the Absence of Vertical Transmission. J Clin Invest (2021) 131(6):1–6. doi: 10.1172/JCI145427 PMC7954587 33497369

[24] Hosier H Farhadian S Morotti RA Deshmukh U Lu-Culligan A Campbell KH . SARS-CoV-2 Infection of the Placenta. JCI (2020) 130(9):4947–53. doi: 10.1172/JCI139569 PMC7456249 32573498

[25] Hecht JL Quade B Deshpande V Mino-Kenudson M Ting DT Desai N . SARS-CoV-2 can Infect the Placenta and Is Not Associated With Specific Placental Histopathology: A Series of 19 Placentas From COVID-19-Positive Mothers. Mod Pathol (2020) 33(11):2092–103. doi: 10.1038/s41379-020-0639-4 PMC7395938 32741970

[26] Facchetti F Bugatti M Drera E Tripodo C Sartori E Cancila V . SARS-CoV2 Vertical Transmission With Adverse Effects on the Newborn Revealed Through Integrated Immunohistochemical, Electron Microscopy and Molecular Analyses of Placenta. EBioMedicine (2020) 59:1–8. doi: 10.1016/j.ebiom.2020.102951 PMC7430280 32818801

[27] Roberts DJ Edlow AG Romero RJ Coyne CB Ting DT Hornick JL . SPECIAL REPORT: A Standardized Definition of Placental Infection by Severe Acute Respiratory Syndrome Coronavirus 2 (SARS-CoV-2), a Consensus Statement From the National Institutes of Health/Eunice Kennedy Shriver National Institute of Child Health and Hu. Am J Obstet Gynecol [Internet] (2021) 2:P593.E1–9. doi: 10.1016/j.ajog.2021.07.029

[28] Tang Z Abrahams VM Mor G Guller S . Placental Hofbauer Cells and Complications of Pregnancy. Ann N Y Acad Sci (2011) 1221:103–8. doi: 10.1111/j.1749-6632.2010.05932.x PMC3707113 21401637

[29] Reyes L Golos TG . Hofbauer Cells: Their Role in Healthy and Complicated Pregnancy. Front Immunol (2018) 9(NOV):1–8. doi: 10.3389/fimmu.2018.02628 30498493 PMC6249321

[30] Lewis SH Fox HE Lewis SH Lewis SH Reynolds-Kohler C Nelson JA . HIV-1 in Trophoblastic and Villous Hofbauer Cells, and Haematological Precursors in Eight-Week Fetuses. Lancet (1990) 335(8689):565–8. doi: 10.1016/0140-6736(90)90349-A 1689792

[31] Backé E Jimenez E Unger M Schafer A Jauniaux E Vogel M . Demonstration of HIV-1 Infected Cells in Human Placenta by *In Situ* Hybridisation and Immunostaining. J Clin Pathol (1992) 45(10):871–4. doi: 10.1136/jcp.45.10.871 PMC495056 1430256

[32] Martin AW Brady K Smith SI DeCoste D Page DV Malpica A . Immunohistochemical Localization of Human Immunodeficiency Virus P24 Antigen in Placental Tissue. Hum Pathol (1992) 23(4 PG-411–4):411–4. doi: 10.1016/0046-8177(92)90088-K 1563742

[33] Backé E Jimenez E Unger M Schäfer A Vocks-Hauck M Grosch-Wörner I . Vertical Human Immunodeficiency Virus Transmission: A Study of Placental Pathology in Relation to Maternal Risk Factors. Am J Perinatol (1994) 11(5):326–30. doi: 10.1055/s-2007-994545 7993509

[34] Sheikh AU Polliotti BM Miller RK . Human Immunedeficiency Virus Infection: *In Situ* Polymerase Chain Reaction Localization in Human Placentas After *In Utero* and *In Vitro* Infection. Am J Obstet Gynecol (2000) 182(1 I PG-207–213):207–13. doi: 10.1016/S0002-9378(00)70514-X 10649180

[35] Bhoopat L Khunamornpong S Sirivatanapa P Rithaporn T Lerdsrimongkol P Thorner PS . Chorioamnionitis Is Associated With Placental Transmission of Human Immunodeficiency Virus-1 Subtype E in the Early Gestational Period. Mod Pathol (2005) 18(10 PG-1357–64):1357–64. doi: 10.1038/modpathol.3800418 15846390

[36] Behbahani H Popek E Garcia P Andersson J Spetz A-L Landay A . Up-Regulation of CCR5 Expression in the Placenta Is Associated With Human Immunodeficiency Virus-1 Vertical Transmission. Am J Pathol (2000) 157(6):1811–8. doi: 10.1016/S0002-9440(10)64819-5 PMC1885789 11106553

[37] Pillay K Cloete M McLeod H . Expression of DC-SIGN and DC-SIGNRs in Placentas of HIV-Positive Patients. South Afr J HIV Med (2014) 15(3 PG-97–101):97–101. doi: 10.4102/sajhivmed.v15i3.8

[38] Martinez J Santiago MR Martelli-Palomino G de Souza DA Silva TGA Silva GEB . Expression of HLA-E Molecules in the Placental Tissue of Women Infected With HIV-1 and Uninfected Women. Placenta (2017) 49:33–6. doi: 10.1016/j.placenta.2016.08.082 28012452

[39] de Noronha L Zanluca C Azevedo MLV Luz KG dos Santos CND . Zika Virus Damages the Human Placental Barrier and Presents Marked Fetal Neurotropism. Mem Inst Oswaldo Cruz (2016) 111(5):287–93. doi: 10.1590/0074-02760160085 PMC4878297 27143490

[40] Rosenberg A Yu W Hill D Reyes C Schwartz D . Placental Pathology of Zika Virus: Viral Infection of the Placenta Induces Villous Stromal Macrophage (Hofbauer Cell) Proliferation and Hyperplasia. Arch Pathol Lab Med (2017) 141(1):43–8. doi: 10.5858/arpa.2016-0401-OA 27681334

[41] Schwartz DA . Viral Infection, Proliferation, and Hyperplasia of Hofbauer Cells and Absence of Inflammation Characterize the Placental Pathology of Fetuses With Congenital Zika Virus Infection. Arch Gynecol Obstet (2017) 295(6):1361–8. doi: 10.1007/s00404-017-4361-5 PMC5429341 28396992

[42] de Noronha L Zanluca C Burger M Suzukawa AA Azevedo M Rebutini PZ . Zika Virus Infection at Different Pregnancy Stages: Anatomopathological Findings, Target Cells and Viral Persistence in Placental Tissues. Front Microbiol (2018) 9(SEP):1–11. doi: 10.3389/fmicb.2018.02266 30337910 PMC6180237

[43] Rabelo K Souza LJ Salomão NG Oliveira ERA Sentinelli L de P Lacerda MS . Placental Inflammation and Fetal Injury in a Rare Zika Case Associated With Guillain-Barré Syndrome and Abortion. Front Microbiol (2018) 9:1018. doi: 10.3389/fmicb.2018.01018 29867903 PMC5964188

[44] Beaufrère A Bessières B Bonnière M Driessen M Alfano C Couderc T . A Clinical and Histopathological Study of Malformations Observed in Fetuses Infected by the Zika Virus. Brain Pathol (2019) 29(1):114–25. doi: 10.1111/bpa.12644 PMC8028325 30020561

[45] Lum FM Narang V Hue S Chen J McGovern N Rajarethinam R . Immunological Observations and Transcriptomic Analysis of Trimester-Specific Full-Term Placentas From Three Zika Virus-Infected Women. Clin Transl Immunol (2019) 8(11):e01082. doi: 10.1002/cti2.1082 PMC6831931 31709049

[46] Santos GR Pinto CAL Prudente RCS Bevilacqua EMAF Witkin SS Passos SD . Histopathologic Changes in Placental Tissue Associated With Vertical Transmission of Zika Virus. Int J Gynecol Pathol (2020) 39(2):157–62. doi: 10.1097/PGP.0000000000000586 30789499

[47] Miranda J Martín-Tapia D Valdespino-Vázquez Y Alarcón L Espejel-Nuñez A Guzmán-Huerta M . Syncytiotrophoblast of Placentae From Women With Zika Virus Infection Has Altered Tight Junction Protein Expression and Increased Paracellular Permeability. Cells (2019) 8(10):P1174. doi: 10.3390/cells8101174 PMC6829373 31569528

[48] Mühlemann K Miller RK Metlay L Menegus MA . Cytomegalovirus Infection of the Human Placenta: An Immunocytochemical Study. Hum Pathol (1992) 23(11):1234–7. doi: 10.1016/0046-8177(92)90290-J 1330874

[49] Schwartz DA Khan R Stoll B . Characterization of the Fetal Inflammatory Response to Cytomegalovirus Placentitis. An Immunohistochemical Study. Arch Pathol Lab Med (1992) 116(1):21–7. 1310378

[50] Mühlemann K Miller RK Metlay L Menegus MA . Characterization of Placental Cytomegalovirus Infection by Immunocytochemistry. Placenta (1994) 15(1):215–22. doi: 10.1016/S0143-4004(05)80345-5

[51] Euscher E Davis J Holzman I Nuovo GJ . Coxsackie Virus Infection of the Placenta Associated With Neurodevelopmental Delays in the Newborn. Obstet Gynecol (2001) 98(6):1019–26. doi: 10.1097/00006250-200112000-00006 11755547

[52] Satosar A Ramirez NC Bartholomew D Davis J Nuovo GJ . Histologic Correlates of Viral and Bacterial Infection of the Placenta Associated With Severe Morbidity and Mortality in the Newborn. Hum Pathol (2004) 35(5):536–45. doi: 10.1016/j.humpath.2004.01.015 15138926

[53] Yao L Korteweg C Hsueh W Gu J . Avian Influenza Receptor Expression in H5N1-Infected and Noninfected Human Tissues. FASEB [Internet] (2008) 22(3):733–40. doi: 10.1096/fj.06-7880com 17925493

[54] Gu J Xie Z Gao Z Liu J Korteweg C Ye J . H5N1 Infection of the Respiratory Tract and Beyond: A Molecular Pathology Study. Lancet (2007) 370(9593):1137–45. doi: 10.1016/S0140-6736(07)61515-3 PMC7159293 17905166

[55] Liu J Feng Y Wang J Li X Lei C Jin D . An “Immune Barrier” Is Formed in the Placenta by Hepatitis B Immunoglobulin to Protect the Fetus From Hepatitis B Virus Infection From the Mother. Hum Vaccin Immunother (2015) 11(8):2068–76. doi: 10.1080/21645515.2015.1010890 PMC4635728 26126021

[56] Ambühl L Leonhard A Widen Zakhary C Jørgensen A Blaakaer J Dybkaer K . Human Papillomavirus Infects Placental Trophoblast and Hofbauer Cells, But Appears Not to Play a Causal Role in Miscarriage and Preterm Labor. Acta Obs Gynecol Scand (2017) 96(10):1188–96. doi: 10.1111/aogs.13190 28699189

[57] Nunes P Nogueira R Coelho J Rodrigues F Salomão N José C . A Stillborn Multiple Organs’ Investigation From a Maternal Denv-4 Infection: Histopathological and Inflammatory Mediators Characterization. Viruses (2019) 11(4):P319. doi: 10.3390/v11040319 PMC6521294 30986974

[58] Salomão N Brendolin M Rabelo K Wakimoto M de Filippis AM Dos Santos F . Spontaneous Abortion and Chikungunya Infection: Pathological Findings. Viruses (2021) 13(4):1–11. doi: 10.3390/v13040554 PMC8067258 33806252

[59] Morotti D Cadamuro M Rigoli E Sonzogni A Gianatti A Parolin C . Molecular Pathology Analysis of SARS-CoV-2 in Syncytiotrophoblast and Hofbauer Cells in Placenta From a Pregnant Woman and Fetus With COVID-19. Pathogens (2021) 10(4):1–13. doi: 10.3390/pathogens10040479 PMC8071113 33920814

[60] Verma S Joshi CS Silverstein RB He M Carter EB Mysorekar IU . SARS-CoV-2 Colonization of Maternal and Fetal Cells of the Human Placenta Promotes Alteration of Local Renin-Angiotensin System. Med [Internet] (2021) 2(5):575–590.e5. doi: 10.1016/j.medj.2021.04.009 PMC8043616 33870242

[61] Gao L Ren J Xu L Ke X Xiong L Tian X . Placental Pathology of the Third Trimester Pregnant Women From COVID-19. Diagn Pathol (2021) 16(1):1–11. doi: 10.1186/s13000-021-01067-6 33441152 PMC7806280

[62] Mano H Chermann JC . Replication of Human Immunodeficiency Virus Type 1 in Primary Cultured Placental Cells. Res Virol [Internet] (1991) 142(2–3):95–104. doi: 10.1016/0923-2516(91)90044-4 1896650

[63] Kesson AM Fear WR Kazazi F Mathijs JM Chang J King NJ . Human Immunodeficiency Virus Type 1 Infection of Human Placental Macrophages *In Vitro* . J Infect Dis [Internet] (1993) 168(3):571–9. doi: 10.1093/infdis/168.3.571 7689088

[64] Kesson AM Fear WR Williams L Chang J King NJC Cunningham AL . HIV Infection of Placental Macrophages: Their Potential Role in Vertical Transmission. J Leukoc Biol (1994) 56(3):241–6. doi: 10.1002/jlb.56.3.241 8083596

[65] McGann KA Collman R Kolson DL Gonzalez-Scarano F Coukos G Coutifaris C . Human Immunodeficiency Virus Type 1 Causes Productive Infection of Macrophages in Primary Placental Cell Cultures. J Infect Dis [Internet] (1994) 169(4):746–53. doi: 10.1093/infdis/169.4.746 8133087

[66] Meléndez-Guerrero L Holmes R Backé E Polliotti B Ibegbu C Lee F . *In Vitro* Infection of Hofbauer Cells With a Monocyte-Tropic Strain of HIV-1. Placenta (1994) 15(1):33–45. doi: 10.1016/S0143-4004(05)80334-0

[67] Lee AW Mitra D Laurence J . Interaction of Pregnancy Steroid Hormones and Zidovudine in Inhibition of HIV Type 1 Replication in Monocytoid and Placental Hofbauer Cells: Implications for the Prevention of Maternal-Fetal Transmission of HIV. AIDS Res Hum Retroviruses (1997) 13(14):1235–42. doi: 10.1089/aid.1997.13.1235 9310291

[68] Fear WR Kesson AM Naif H Lynch GW Cunningham AL . Differential Tropism and Chemokine Receptor Expression of Human Immunodeficiency Virus Type 1 in Neonatal Monocytes, Monocyte-Derived Macrophages, and Placental Macrophages. J Virol (1998) 72(2):1334–44. doi: 10.1128/JVI.72.2.1334-1344.1998 PMC124612 9445034

[69] Plaud-Valentin M Delgado R Garcia V Zorrilla C Gandia J Meléndez-Guerrero L . HIV Infection of Placental Macrophages: Effect on the Secretion of HIV Stimulatory Cytokines. Cell Mol Biol (1999) 45(4):423–31. 10432189

[70] Bácsi A Csoma E Beck Z Andirkó I Kónya J Gergely L . Induction of HIV-1 Replication in Latently Infected Syncytiotrophoblast Cells by Contact With Placental Macrophages: Role of Interleukin-6 and Tumor Necrosis Factor-Alpha. J Interferon Cytokine Res (2001) 21(12 PG-1079–88):1079–88. doi: 10.1089/107999001317205213 11798466

[71] Luciano-Montalvo C Ciborowski P Duan F Gendelman HE Meléndez LM . Proteomic Analyses Associate Cystatin B With Restricted HIV-1 Replication in Placental Macrophages. Placenta (2008) 29(12):1016–23. doi: 10.1016/j.placenta.2008.09.005 PMC3867668 18951626

[72] Luciano-Montalvo C Meléndez LM . Cystatin B Associates With Signal Transducer and Activator of Transcription 1 in Monocyte-Derived and Placental Macrophages. Placenta (2009) 30(5):464–7. doi: 10.1016/j.placenta.2009.03.003 PMC2683624 19342095

[73] García-Crespo K Cadilla C Skolasky R Meléndez LM . Restricted HIV-1 Replication in Placental Macrophages Is Caused by Inefficient Viral Transcription. J Leukoc Biol (2010) 87(4):633–6. doi: 10.1189/jlb.0809556 PMC2858303 20042472

[74] Boily-Larouche G Milev MP Zijenah LS Labbé AC Zannou DM Humphrey JH . Naturally-Occurring Genetic Variants in Human DC-SIGN Increase HIV-1 Capture, Cell-Transfer and Risk of Mother-to-Child Transmission. PloS One (2012) 7(7):e40706. doi: 10.1371/journal.pone.0040706 22808239 PMC3393705

[75] Johnson EL Chakraborty R . Placental Hofbauer Cells Limit HIV-1 Replication and Potentially Offset Mother to Child Transmission (MTCT) by Induction of Immunoregulatory Cytokines. Retrovirology (2012) 9(101):1–11. doi: 10.1186/1742-4690-9-101 23217137 PMC3524025

[76] Johnson EL Chu H Byrareddy SN Spearman P Chakraborty R . Placental Hofbauer Cells Assemble and Sequester HIV-1 in Tetraspanin-Positive Compartments That Are Accessible to Broadly Neutralizing Antibodies. J Int AIDS Soc (2015) 18(1):1–8. doi: 10.7448/IAS.18.1.19385 PMC4308659 25623930

[77] Johnson EL Boggavarapu S Johnson ES Lal AA Agrawal P Bhaumik SK . Human Cytomegalovirus Enhances Placental Susceptibility and Replication of Human Immunodeficiency Virus Type 1 (HIV-1), Which May Facilitate *In Utero* HIV-1 Transmission. J Infect Dis (2018) 218(9):1464–73. doi: 10.1093/infdis/jiy327 PMC6927849 29860306

[78] El Costa H Gouilly J Mansuy JM Chen Q Levy C Cartron G . ZIKA Virus Reveals Broad Tissue and Cell Tropism During the First Trimester of Pregnancy. Sci Rep (2016) 6(PG-35296):35296. doi: 10.1038/srep35296 27759009 PMC5069472

[79] Jurado K Simoni MK Tang Z Uraki R Hwang J Householder S . Zika Virus Productively Infects Primary Human Placenta-Specific Macrophages. JCI Insight (2016) 1(13):1–6. doi: 10.1172/jci.insight.88461 PMC5007065 27595140

[80] Tabata T Petitt M Puerta-Guardo H Michlmayr D Wang C Fang-Hoover J . Zika Virus Targets Different Primary Human Placental Cells, Suggesting Two Routes for Vertical Transmission. Cell Host Microbe (2016) 20(2):155–66. doi: 10.1016/j.chom.2016.07.002 PMC5257282 27443522

[81] Quicke KM Bowen JR Johnson EL McDonald CE Ma H O’Neal JT . Zika Virus Infects Human Placental Macrophages. Cell Host Microbe (2016) 20(1 PG-83–90):83–90. doi: 10.1016/j.chom.2016.05.015 27247001 PMC5166429

[82] Tabata T Petitt M Puerta-Guardo H Michlmayr D Harris E Pereira L . Zika Virus Replicates in Proliferating Cells in Explants From First-Trimester Human Placentas, Potential Sites for Dissemination of Infection. J Infect Dis (2018) 217(8):1202–13. doi: 10.1093/infdis/jix552 PMC6075529 29106643

[83] Gavegnano C Bassit LC Cox BD Hsiao H-M Johnson EL Suthar M . Jak Inhibitors Modulate Production of Replication-Competent Zika Virus in Human Hofbauer, Trophoblasts, and Neuroblastoma Cells. Pathog Immun (2017) 2(2):199–218. doi: 10.20411/pai.v2i2.190 28776046 PMC5538373

[84] Zimmerman MG Quicke KM O’Neal JT Arora N Machiah D Priyamvada L . Cross-Reactive Dengue Virus Antibodies Augment Zika Virus Infection of Human Placental Macrophages. Cell Host Microbe (2018) 24(5):731–42. doi: 10.1016/j.chom.2018.10.008 PMC6394860 30439342

[85] Puerta-Guardo H Tabata T Petitt M Dimitrova M Glasner DR Pereira L . Zika Virus Nonstructural Protein 1 Disrupts Glycosaminoglycans and Causes Permeability in Developing Human Placentas. J Infect Dis (2020) 221(2):313–24. doi: 10.1093/infdis/jiz331 PMC6936002 31250000

[86] Bácsi A Aranyosi J Beck Z Ebbesen P Andirkó I Szabó J . Placental Macrophage Contact Potentiates the Complete Replicative Cycle of Human Cytomegalovirus in Syncytiotrophoblast Cells: Role of Interleukin-8 and Transforming Growth Factor-β1. J Interf Cytokine Res (1999) 19(10 PG-1153–1160):1153–60. doi: 10.1089/107999099313091 10547155

[87] Wussow F Chiuppesi F Martinez J Campo J Johnson E Flechsig C . Human Cytomegalovirus Vaccine Based on the Envelope Gh/gL Pentamer Complex. PloS Pathog (2014) 10(11):e1004524. doi: 10.1371/journal.ppat.1004524 25412505 PMC4239111

[88] Plaeger-Marshall S Ank BJ Altenburger KM Pizer LI Johnston RB Stiehm ER . Replication of Herpes Simplex Virus in Blood Monocytes and Placental Macrophages From Human Neonates. Pediatr Res (1989) 26(2):135–9. doi: 10.1203/00006450-198908000-00014 2549493

[89] Oliveira LHS Fonseca MEF De Bonis M . Placental Phagocytic Cells Infected With Herpes Simplex Type 2 and Echovirus Type 19: Virological and Ultrastructural Aspects. Placenta (1992) 13(5):405–16. doi: 10.1016/0143-4004(92)90048-X 1335148

[90] Toth FD Norskov-Lauritsen N Juhl CB Aboagye-Mathiesen G Ebbesen P . Interferon Production by Cultured Human Trophoblasts and Choriocarcinoma Cell Lines Induced by Sendai Virus. J Gen Virol (1990) 71(12):3067–9. doi: 10.1099/0022-1317-71-12-3067 2177096

[91] Bokun V Moore JJ Moore R Smallcombe CC Harford TJ Rezaee F . Respiratory Syncytial Virus Exhibits Differential Tropism for Distinct Human Placental Cell Types With Hofbauer Cells Acting as a Permissive Reservoir for Infection. PloS One (2019) 14(12 PG-e0225767):1–16. doi: 10.1371/journal.pone.0225767 PMC6886783 31790466

[92] Hendrix P Tang Z Silasi M Racicot KE Mor G Abrahams VM . Herpesvirus-Infected Hofbauer Cells Activate Endothelial Cells Through an IL-1β-Dependent Mechanism. Placenta (2020) 91:59–65. doi: 10.1016/j.placenta.2020.01.010 32174308 PMC7078070

[93] Lu-Culligan A Chavan AR Vijayakumar P Irshaid L Courchaine EM Milano KM . SARS-CoV-2 Infection in Pregnancy is Associated With Robust Inflammatory Response at The Maternal-Fetal Interface. Medrxiv : The Preprint Server for Health Sciences. (2021). doi: 10.1101/2021.01.25.21250452 PMC8084634 33969332

[94] Atwood WJ Berger JR Kaderman R Tornatore CS Major EO . Human Immunodeficiency Virus Type 1 Infection of the Brain. Clin Microbiol Rev (1993) 6(4):339–66. doi: 10.1128/CMR.6.4.339 PMC358293 8269391

[95] Hennessey M Fischer M Staples JE . Zika Virus Spreads to New Areas - Region of the Americas, May 2015-January 2016. MMWR Morb Mortal Wkly Rep (2016) 65(3):55–8. doi: 10.15585/mmwr.mm6503e1 26820163

[96] Soares F Abranches AD Villela L Lara S Araújo D Nehab S . Zika Virus Infection in Pregnancy and Infant Growth, Body Composition in the First Three Months of Life: A Cohort Study. Sci Rep (2019) 9(1):6–11. doi: 10.1038/s41598-019-55598-6 31844129 PMC6915782

[97] Krauer F Riesen M Reveiz L Oladapo OT Martínez-Vega R Porgo TV . Zika Virus Infection as a Cause of Congenital Brain Abnormalities and Guillain–Barré Syndrome: Systematic Review. PloS Med (2017) 14(1):1–27. doi: 10.1371/journal.pmed.1002203 PMC5207634 28045901

[98] Doyle RM Harris K Kamiza S Harjunmaa U Ashorn U Nkhoma M . Bacterial Communities Found in Placental Tissues Are Associated With Severe Chorioamnionitis and Adverse Birth Outcomes. PloS One (2017) 12(7):1–23. doi: 10.1371/journal.pone.0180167 PMC5507499 28700642

[99] Tkachuk AN Moormann AM Poore JA Rochford RA Chensue SW Mwapasa V . Malaria Enhances Expression of CC Chemokine Receptor 5 on Placental Macrophages. J Infect Dis (2001) 183(6):967–72. doi: 10.1086/319248 11237815

[100] Chaisavaneeyakorn S Lucchi N Abramowsky C Othoro C Chaiyaroj SC Shi YP . Immunohistological Characterization of Macrophage Migration Inhibitory Factor Expression in Plasmodium Falciparum-Infected Placentas. Infect Immun (2005) 73(6):3287–93. doi: 10.1128/IAI.73.6.3287-3293.2005 PMC1111854 15908353

[101] Gaw SL Hromatka BS Ngeleza S Buarpung S Ozarslan N Tshefu A . Differential Activation of Fetal Hofbauer Cells in Primigravidas Is Associated With Decreased Birth Weight in Symptomatic Placental Malaria. Malar Res Treat (2019) 2019:1–10. doi: 10.1155/2019/1378174 PMC6521392 31186834

[102] Rindsjö E Varli IH Ofori MF Lundquist M Holmlund U Papadogiannakis N . Presence of IgE+ Cells in Human Placenta Is Independent of Malaria Infection or Chorioamnionitis. Clin Exp Immunol (2006) 144(2):204–11. doi: 10.1111/j.1365-2249.2006.03055.x PMC1809662 16634792

[103] Walter P Blot P Ivanoff B . The Placental Lesions in Congenital Syphilis - A Study of Six Cases. Virchows Arch A Pathol Anat Histol (1982) 397(3):313–26. doi: 10.1007/BF00496572 7157668

[104] Amara AB Gorvel L Baulan K Derain-Court J Buffat C Vérollet C . Placental Macrophages Are Impaired in Chorioamnionitis, an Infectious Pathology of the Placenta. J Immunol (2013) 191(11):5501–14. doi: 10.4049/jimmunol.1300988 24163411

[105] Doster RS Sutton JA Rogers LM Aronoff DM Gaddy JA . Streptococcus Agalactiae Induces Placental Macrophages To Release Extracellular Traps Loaded With Tissue Remodeling Enzymes *via* an Oxidative Burst-Dependent Mechanism. MBio (2018) 9(6):1–16. doi: 10.1128/mBio.02084-18 PMC6247082 30459195

[106] Sutton JA Rogers LM Dixon B Kirk L Doster R Holly AM . Protein Kinase D Mediates Inflammatory Responses of Human Placental Macrophages to Group B Streptococcus. Am J Reprod Immunol (2019) 81(3 PG-e13075):1–22. doi: 10.1111/aji.13075 PMC6459189 30582878

[107] Mason KL Rogers LM Soares EM Bani-Hashemi T Erb Downward J Agnew D . Intrauterine Group A Streptococcal Infections Are Exacerbated by Prostaglandin E 2. J Immunol (2013) 191(5):2457–65. doi: 10.4049/jimmunol.1300786 PMC3750066 23913961

[108] Rogers LM Anders AP Doster RS Gill EA Gnecco JS Holley JM . Decidual Stromal Cell-Derived PGE2 Regulates Macrophage Responses to Microbial Threat. Am J Reprod Immunol (2018) 80(4):e13032. doi: 10.1111/aji.13032 30084522 PMC6461368

[109] Mezouar S Benammar I Boumaza A Diallo AB Chartier C Buffat C . Full-Term Human Placental Macrophages Eliminate Coxiella Burnetii Through an IFN-γ Autocrine Loop. Front Microbiol (2019) 10:1–12. doi: 10.3389/fmicb.2019.02434 31749776 PMC6842979

[110] Thomas J Appios A Zhao X Dutkiewicz R Donde M Lee C . Phenotypic and Functional Characterisation of First Trimester Human Placental Macrophages, Hofbauer Cells. J Exp Med (2020) 218(1):1–17. doi: 10.1084/jem.20200891 PMC7579740 33075123

[111] Pavlov O Pavlova O Ailamazyan E Selkov S . Characterization of Cytokine Production by Human Term Placenta Macrophages *In Vitro* . Am J Reprod Immunol (2008) 60(6):556–67. doi: 10.1111/j.1600-0897.2008.00657.x 18853988

[112] Pavlov OV Niauri DA Selutin AV Selkov SA . Coordinated Expression of Tnfα- and VEGF-Mediated Signaling Components by Placental Macrophages in Early and Late Pregnancy. Placenta (2016) 42:28–36. doi: 10.1016/j.placenta.2016.04.008 27238711

[113] Schliefsteiner C Ibesich S Wadsack C . Placental Hofbauer Cell Polarization Resists Inflammatory Cues *In Vitro* . Int J Mol Sci (2020) 21(3):1–14. doi: 10.3390/ijms21030736 PMC7038058 31979196

[114] Young O Tang Z Niven-Fairchild T Tadesse S Krikun G Norwitz E . Toll-Like Receptor-Mediated Responses by Placental Hofbauer Cells (HBCs): A Potential Pro-Inflammatory Role for Fetal M2 Macrophages. Am J Reprod Immunol (2015) 73(1):22–35. doi: 10.1111/aji.12336 25345551 PMC4268350

[115] Abrahams VM Tang Z Mor G Guller S . NLRP3 Inflammasome Function and Pyroptotic Cell Death in Human Placental Hofbauer Cells. J Reprod Immunol (2020) 142(July):103214. doi: 10.1016/j.jri.2020.103214 33152658 PMC7770077

[116] May K Grube M Malhotra I Long CA Singh S Mandaliya K . Antibody-Dependent Transplacental Transfer of Malaria Blood-Stage Antigen Using a Human *Ex Vivo* Placental Perfusion Model. PloS One (2009) 4(11):e7986. doi: 10.1371/journal.pone.0007986 19956710 PMC2777305

[117] Urbanelli L Buratta S Tancini B Sagini K Delo F Porcellati S . The Role of Extracellular Vesicles in Viral Infection and Transmission. Vaccines (2019) 7(3):1–20. doi: 10.3390/vaccines7030102 PMC6789493 31466253

[118] Duval C Brien ME Gaudreault V Boufaied I Baker B Jones RL . Differential Effect of LPS and IL-1β in Term Placental Explants. Placenta (2019) 75(October 2018):9–15. doi: 10.1016/j.placenta.2018.11.006 30712669

[119] Castellucci M Kaufmann P . A Three-Dimensional Study of the Normal Human Placental Villous Core - I. The Hofbauer Cells. Placenta (1982) 210(3):269–85. doi: 10.1016/S0143-4004(82)80004-0 7134195

