# Supplementary material for: Placental Macrophage (Hofbauer Cell) Responses to Infection During Pregnancy: A Systematic Scoping Review
Source: Front Immunol. 2022 Feb 11;12:756035. doi: 10.3389/fimmu.2021.756035 (PMC8895398; doi:10.3389/fimmu.2021.756035)
Supplement: Supplementary file 1 [file DataSheet_1.docx]

# **Supplementary material**

**Table 1:** Keywords added in the final search strategy without obtaining any new results

| To capture studies of infections | | To capture responses to PAMPs | |
| --- | --- | --- | --- |
| Rubella | CMV | PAMP* | "Polyic" |
| Syphilis | VZV | "Pathogen-associated molecular pattern" | "Poly(ic)" |
| Malaria | Ebola | "Pathogen associated molecular patterns" | "Poly (ic)" |
| Chlamydia | Hepatitis | PRR* | "Polyinosinic:polycytidylic acid" |
| Streptococc* | Fungi | "Pattern recognition receptors" | "Polyinosinic: polycytidylic acid" |
| "TORCH" | Fungus | "Pattern recognition receptor" |  |
| "TORCH syndrome" | Fungal | "Poly (i:c)" |  |
| SARS-CoV2 | COVID-19 | "Poly i:c" |  |
| Coronavirus | COVID19 | "Polyi:c" |  |
| ZIKV |  |  |  |

**Table 2:** Title and source of studies without full text available

|  |  |
| --- | --- |
| Title | **Source (PMID)** |
| Immunohistochemical study of the morphological changes in placental villi from fetal membranes infectious disease | PubMed (17983186) |
| Histopathological Study of Human Placenta in Women Infected With Rubella Virus During Pregnancy | PubMed (144235) |
| Is Decreased HIV-1 Infectivity of Placental Macrophages Caused by High Levels of Beta-Chemokines? | PubMed (11936874) |
| Piacental (*sic*) blockade of maternal fetal HIV transmission is breached wtth (*sic*) low maternal cd4 percent and high viral load | Scopus |
